# Establishment and validation of an aging-related risk signature associated with prognosis and tumor immune microenvironment in breast cancer

**DOI:** 10.1186/s40001-022-00924-4

**Published:** 2022-12-29

**Authors:** Zitao Wang, Hua Liu, Yiping Gong, Yanxiang Cheng

**Affiliations:** 1 Department of Obstetrics and Gynecology, Renmin Hospital of Wuhan University, Wuhan, Hubei China; 2 Department of Breast Surgery, Renmin Hospital of Wuhan University, Wuhan, Hubei China

**Keywords:** Breast cancer (BC), Aging-related genes (ARGs), Survival, Tumor immune microenvironment, Immunotherapy

## Abstract

**Background:**

Breast cancer (BC) is a highly malignant and heterogeneous tumor which is currently the cancer with the highest incidence and seriously endangers the survival and prognosis of patients. Aging, as a research hotspot in recent years, is widely considered to be involved in the occurrence and development of a variety of tumors. However, the relationship between aging-related genes (ARGs) and BC has not yet been fully elucidated.

**Materials and methods:**

The expression profiles and clinicopathological data were acquired in the Cancer Genome Atlas (TCGA) and the gene expression omnibus (GEO) database. Firstly, the differentially expressed ARGs in BC and normal breast tissues were investigated. Based on these differential genes, a risk model was constructed composed of 11 ARGs via univariate and multivariate Cox analysis. Subsequently, survival analysis, independent prognostic analysis, time-dependent receiver operating characteristic (ROC) analysis and nomogram were performed to assess its ability to sensitively and specifically predict the survival and prognosis of patients, which was also verified in the validation set. In addition, functional enrichment analysis and immune infiltration analysis were applied to reveal the relationship between the risk scores and tumor immune microenvironment, immune status and immunotherapy. Finally, multiple datasets and real‐time polymerase chain reaction (RT-PCR) were utilized to verify the expression level of the key genes.

**Results:**

An 11-gene signature (*including FABP7*, *IGHD*, *SPIB*, *CTSW*, *IGKC*, *SEZ6*, *S100B*, *CXCL1*, *IGLV6-57*, *CPLX2* and *CCL19*) was established to predict the survival of BC patients, which was validated by the GEO cohort. Based on the risk model, the BC patients were divided into high- and low-risk groups, and the high-risk patients showed worse survival. Stepwise ROC analysis and Cox analyses demonstrated the good performance and independence of the model. Moreover, a nomogram combined with the risk score and clinical parameters was built for prognostic prediction. Functional enrichment analysis revealed the robust relationship between the risk model with immune-related functions and pathways. Subsequent immune microenvironment analysis, immunotherapy, etc., indicated that the immune status of patients in the high-risk group decreased, and the anti-tumor immune function was impaired, which was significantly different with those in the low-risk group. Eventually, the expression level of *FABP7*, *IGHD*, *SPIB*, *CTSW*, *IGKC*, *SEZ6*, *S100B*, *CXCL1*, *IGLV6-57* and *CCL19* was identified as down-regulated in tumor cell line, while CPLX2 up-regulated, which was mostly similar with the results in TCGA and Human Protein Atlas (HPA) via RT-PCR.

**Conclusions:**

In summary, our study constructed a risk model composed of ARGs, which could be used as a solid model for predicting the survival and prognosis of BC patients. Moreover, this model also played an important role in tumor immunity, providing a new direction for patient immune status assessment and immunotherapy selection.

**Supplementary Information:**

The online version contains supplementary material available at 10.1186/s40001-022-00924-4.

## Introduction

BC is the most common malignant tumor in women, and is also the leading cause of cancer death among women. The number of new cases of BC worldwide reached 2.26 million in 2020, making it the world’s most common cancer, accounting for 24.5% of all new cases of cancer among women, and the number of deaths among women reached 680,000, ranking first in the world. In China, there were 420,000 new cases of female BC in 2020, ranking first among all female malignant tumors [1–3]. With the rapid development of precision medicine, the early diagnosis technology and clinical treatment of BC have also made cutting-edge success and major breakthroughs. However, the mortality rate of BC remains high and the incidence of BC is gradually getting younger, which is a common cause of death in women under 45. What’s more important, patients with different subtypes of BC respond differently to treatment regimens. For example, highly aggressive triple negative BC lacks specificity for endocrine therapy and molecular targeted therapy, contributing to the poor prognosis and early metastasis. Moreover, even if early treatment is effective for BC, about 30% of patients may recur and metastasize, more than 6–7% of new BC is diagnosed as advanced BC [4–6]. Meanwhile, BC is an inherited, highly heterogeneous disease [7] with a complex mechanism of carcinogenesis involving multiple genetic and epigenetics changes [8]. Therefore, it is very important to develop more efficient and sensitive early diagnostic techniques and biomarkers for predicting prognosis.

Aging is an inevitable time-dependent biological process and a common feature of biological organisms. With the passage of time, the gradual accumulation of biological changes within the cell, the susceptibility to diseases such as cancer has gradually increased [9]. In cancer research, cancer and aging are seen as two sides of the same underlying cell and molecule, it is now generally accepted that aging is an independent risk factor for many chronic diseases such as cardiovascular diseases [10], neurodegeneration [11] and malignant tumors [12] such as rectal cancer and lung cancer, which is likely to promote carcinogenesis, tumor progression and cancer treatment resistance. But at the same time, aging is also defined as irreversible growth arrest, resulting in the inhibition of uncontrolled proliferation of tumor cells, which is extremely complex [13]. Therefore, the most important challenge is to analyze the correlation between candidate aging markers and their ability to predict the development and treatment of tumors. Recently, the use of ARGs as a biomarker for diagnosis or prognosis has attracted the attention of researchers in the field of oncology, providing a sensitive and efficient indicator and direction for the diagnosis and treatment of tumors. However, the prognosis of ARGs and its biological function in BC are still unclear.

In this study, we constructed a prognostic risk model consisting of 11 ARGs based on datasets from public databases to assess and predict survival outcomes, clinical characteristics, gene mutations, and immune microenvironment in BC patients. To some extent, it reveals the potential mechanism of BC and provides a new direction for the treatment of BC.

## Materials and methods

### Dataset acquisition

The transcriptional expression profiles and clinical characteristics of BC patients were obtained from the Cancer Genome Atlas (TCGA) (https://portal.gdc.cancer.gov) and GEO (GSE158309) (https://www.ncbi.nlm.nih.gov/geo/) databases, which contained 1109 BC tissues and 113 normal breast tissues in TCGA, as well as 327 patients in GEO (Table 1).

**Table 1:** Clinical parameters of the TCGA and GEO cohort

Characteristics	TCGA cohort (N = 1076)	GEO cohort (N = 327)
Age (years)		
≤ 65	773 (71.84)	305 (93.27)
> 65	303 (28.16)	22 (6.73)
Gender		
Female	1064 (98.88)	327 (100)
Male	12 (1.12)	
T classification		
T1	281 (26.12)	101 (30.89)
T2	621 (57.71)	188 (57.49)
T3	133 (12.36)	26 (7.95)
T4	38 (3.53)	12 (3.67)
NA	3 (0.28)	
N classification		
N0	504 (46.84)	137 (41.90)
N1	361 (33.55)	87 (26.61)
N2	120 (11.15)	63 (19.27)
N3	74 (6.88)	40 (12.22)
NA	17 (1.58)	
M classification		
M0	895 (83.18)	319 (97.55)
M1	22 (2.04)	8 (2.45)
NA	159 (14.78)	
Stage		
St1	183 (17.01)	
St2	608 (56.51)	
St3	242 (22.49)	
St4	24 (2.23)	
NA	23 (1.76)	

### Identification of differentially expressed aging-related genes (ARGs)

A total of 307 human ARGs (Additional file 4: Table S1) were obtained from the Human Aging Genome Resource 3. After extracting the expression files of ARGs, *P* < 0.05 was considered as screening condition to show the differential expression of ARGs and transcription factors (TF) via “Limma”. Stepwise analysis such as functional enrichment analysis and identification of WGCNA modules were employed to reveal the potential biological function of differentially expressed ARGs and the regulatory network of TFs, which were displayed in a heatmap via the “heatmap” package.

### Consensus cluster analysis

Consensus cluster analysis was carried out to classify patients into different group to ensure maximum difference between groups and minimum difference within groups via “ConsensusClusterPlus”. Additionally, the R packages “Survival” and “Survminer” are used to analyze the correlation between the cluster and the overall survival (OS), which was then presented as a Kaplan–Meier (KM) curve.

### Establishment of a risk model based on the prognostic ARGs

We assigned 1076 patients with TCGA to the training set and 327 patients with GEO to the testing set. First and forest, the risk model was constructed via Lasso regression and SVM based on the training set. The obtained genes and the corresponding regression coefficients were used to calculate the specific risk score of each patient. Therefore, patients were divided into high-risk group and low-risk group according to the median of risk score. Moreover, the KM survival curve and the receiver operating characteristic curve were plotted. Eventually, as an external validation set, the risk score of each patient based on the risk signature was calculated to verify the validity of the risk model in the GEO dataset.

### Independent prognostic analysis and construction of the nomogram

To explore whether risk signature was an independent prognostic factor, we combined clinical characteristics of TCGA patients with risk score and analyzed them via univariate and multivariate analyses. Besides, a nomogram integrating the risk score and other clinical parameters was established in training set. In order to evaluate the predictive sensitivity and specificity of the model, we also performed the calibration curves at 1-, 3- and 5-year survival.

### Gene set enrichment analysis

Gene Enrichment Analysis (GSEA) is a computational method used to determine functional differences between two groups. The enrichment of KEGG and GO pathway in high-risk and low-risk groups was analyzed by GSEA (version 4.1.0).

### Mutation analysis

Further analysis of mutation data in BC patients using the “maftools” package which derived from TCGA was processed. We then identified and analyzed the frequency and magnitude of gene mutation spectrum in the patients, compared different gene mutations in the high-and low-risk groups, and calculated the tumor mutation load (TMB) score for each patient. Besides, TMB score and its correlation between groups were also analyzed.

### Evaluation of immune infiltrating cells

CIBERSORT is a deconvolution algorithm based on RNA-Seq data for estimating the composition and abundance of immune cells. Based on the BC dataset, we calculated the relative proportions of 21 types of infiltrating immune cells in all tumor samples, and investigated the differences in infiltrating levels between high-risk and low-risk groups. And the survival analysis of immune cells in high- and low-risk group was applied to identify the relationship between immune cells and survival prognosis of patients. Meanwhile, 11 key genes in the risk model were explored and evaluated in the TIMER database.

### Evaluation of immune status

Single-sample GSEA (ssGSEA) was performed to calculate the enrichment scores of 16 infiltrating immune cells and the activity of 13 immune-related pathways in the high-and low-risk groups using the “GSVA” package. The relationship between the concentration of immune cells and immune-related pathway and survival prognosis was also studied.

### Tumor immune landscape

To investigate the relationship between tumor microenvironment and risk score, ESTIMATE was used to calculate the score of each sample, including tumor purity, ESTIMATE score, immune score, and stromal score.

### Evaluation of immunotherapy

TIDE is currently the best predictors of ANTI-PD1 and CTLA4 therapy, so we estimated the outcome of immunotherapy in high- and low-risk groups based on TIDE scores, which was listed in the form of a violin map. Furthermore, we evaluated and compared the predictive power of our risk model with the TIDE and TIS models.

### GeneMANIA

The GeneMANIA site (https://GeneMANIA.org) is used to predict the functional similarity of the hub genes and to construct a PPI network between them. It also predicts the relationships between functionally similar genes and pivotal genes, including protein–protein, protein-DNA interactions, pathways, physiological and biochemical reactions, co-expression, and co-localization. In this study, we explored the functional similarity of pivotal genes and performed functional enrichment analysis.

### Verification of gene expression level in the risk model

The gene expression profiling database (GEPIA) was used to demonstrate the mRNA expression levels of characteristic genes in BC and normal breast tissues. Immunohistochemical results were obtained for the corresponding genes from the HPA (https://www.proteinatlas.org) to elucidate the protein expression levels of these genes.

### RT-PCR

The expression of the ARGs in the risk signature was validated by real‐time polymerase chain reaction (RT–PCR) using HC11 cell line and 4T1 cell line. Total RNA was extracted from cells using TRIzol reagent and reverse transcribed into cDNA using reverse transcriptase according to protocol provided by the manufacturer. GAPDH was considered as control group according to the 2^−ΔΔCT^ method. All quantitative PCRs were conducted in triplicate.

### Statistical analysis

Student’s *t*-test was used to compare gene expression between tumor cell line and normal cell line. Kaplan–Meier analysis was used to determine the independent risk factors of OS by log rank test. The ROC curve was used to evaluate the diagnostic value of risk score and nomogram. All statistical analyses were performed using R software (version 3.6.1). *P* < 0.05 was considered to be statistically significant.

## Results

### Screening aging-related differentially expressed genes (DEGs)

**Fig. 1:**
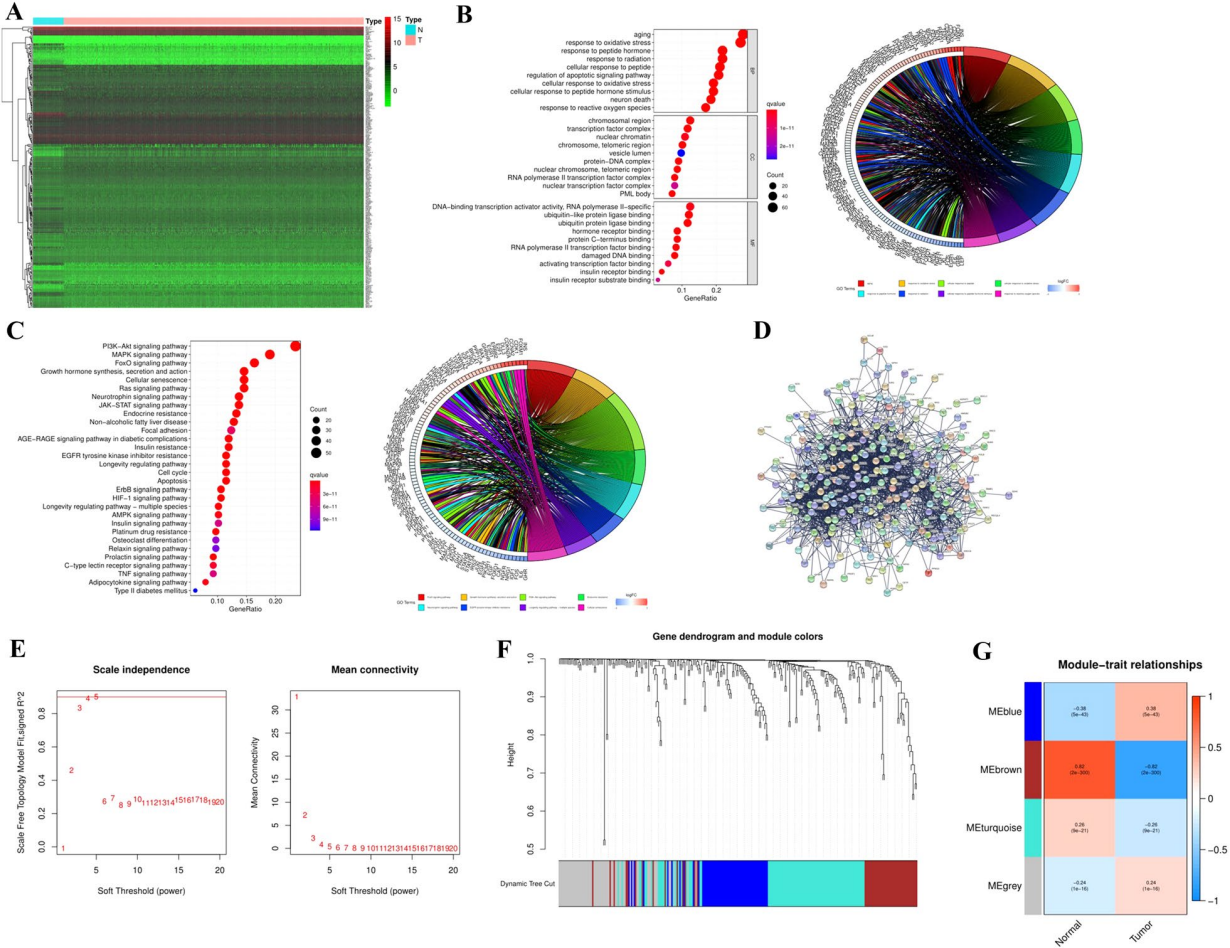
Identification and exploration of differentially expressed ARGs. **A** The differentially expressed genes were shown in a heatmap (green: low expression level; red: high expression). **B** GO enrichment analysis of DEGs. **C** KEGG enrichment analysis of DEGs. **D** Co-expression analysis in PPI network. **E** The threshold to determine the relative balance between scale independence and average connectivity in WGCNA. **F**, **G** Four modules generated by hierarchical average linkage clustering

According to the ARGs obtained from public database, 253 DEGs were acquired by comparing tumor tissues with normal tissues, including 125 down-regulated genes and 128 up-regulated genes (*P* < 0.05, Fig. 1A). In order to further analyze the enriched function by the DEGs, GO and KEGG analysis were performed. The results of GO enrichment analysis showed that these DEGs were closely related to aging, oxidative stress, apoptosis and transcription factor complex (Fig. 1B). In addition, enrichment analysis of the KEGG pathway indicated that there were enriched in cell senescence, apoptosis and classical pathways such as PI3K/AKT signaling pathway (Fig. 1C). Stepwise, STRING network was employed to describe networks of DEGs in Fig. 1D. Next, we determined and removed outliers in each sample, and then performed hierarchical clustering. In WGCNA analysis, we chose the soft threshold to determine the relative balance between scale independence and average connectivity. As shown in Fig. 1E, power = 5 was the threshold, and then four modules were generated by means of hierarchical average linkage clustering (Fig. 1F). ME brown was the most relevant to BC, and ME grey is the least associated with BC (Fig. 1G).

### Clustering analysis based on DEGs

**Fig. 2:**
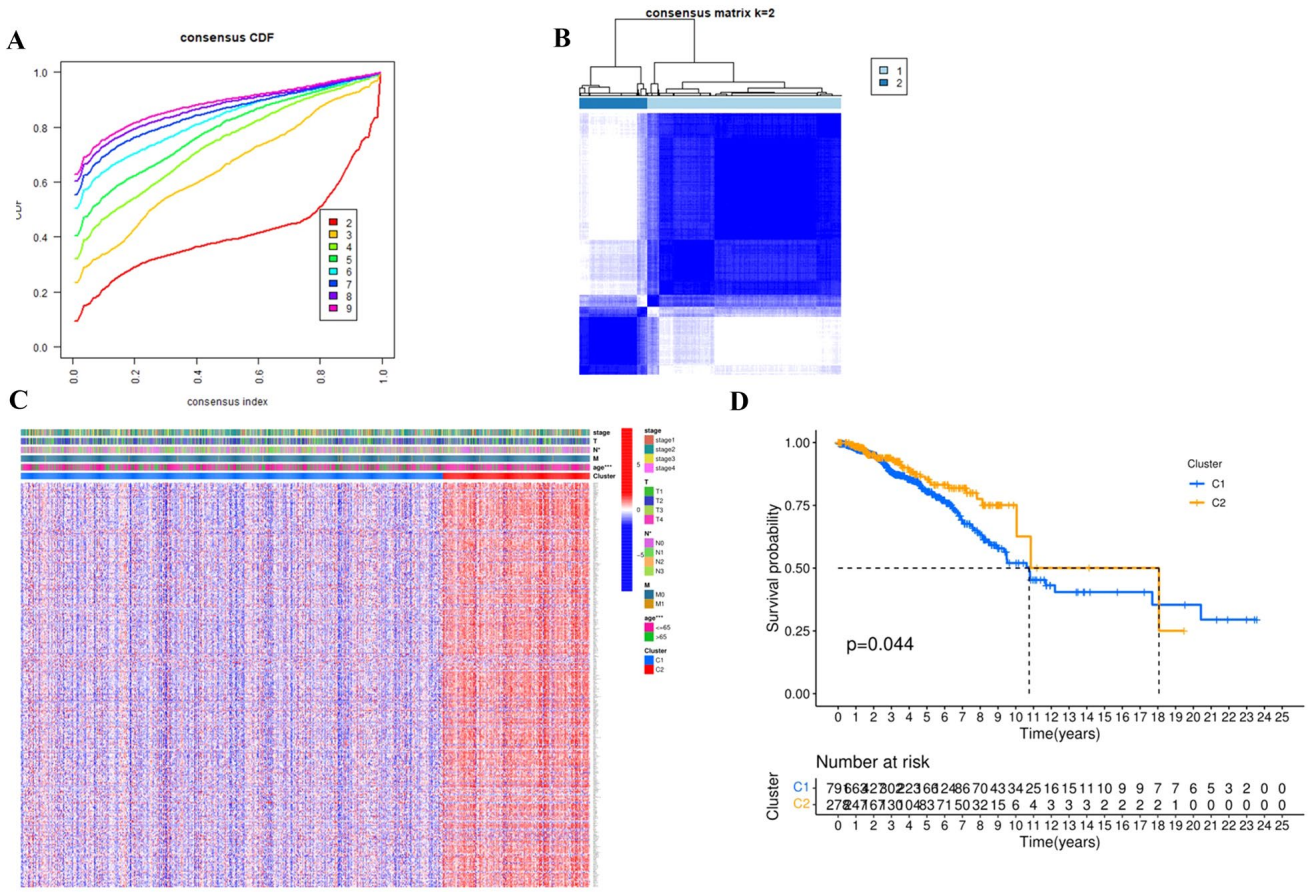
Consensus clustering analysis based on DEGs. **A**, **B** 1076 BC patients were divided into two groups according to the consensus clustering matrix (k = 2). **C** The heatmap illustrated the gene expression profiles and clinical features including stage, T, N, M and age. **D** Kaplan–Meier OS curves between the two groups

To investigate the relationship between BC subtypes and ARG expression, hierarchical cluster analysis was performed in TCGA. By increasing the cluster variable (K) from 2 to 10, we found that when k = 2, the intra-group correlation was highest and the inter-group correlation was lowest, indicating that 1076 BC patients could be well divided into two groups (Fig. 2A, B). The gene expression profiling and clinical features including T (T1, T2, T3 and T4), N (N0, N1, N2 and n3), M (M0 and M1), Stage (Stage 1, 2, 3 and 4) and age (≤ 65 or > 65) were shown in the heatmap, and we found that in both groups, clinical features such as N staging (*P* < 0.05) and age (*P* < 0.001) were significantly different (Fig. 2C). Further survival analysis between the two groups showed that the prognosis of C1 group was significantly worse than C2 group (*P* < 0.05) (Fig. 2D).

### Establishment of prognostic risk model in TCGA

**Fig. 3:**
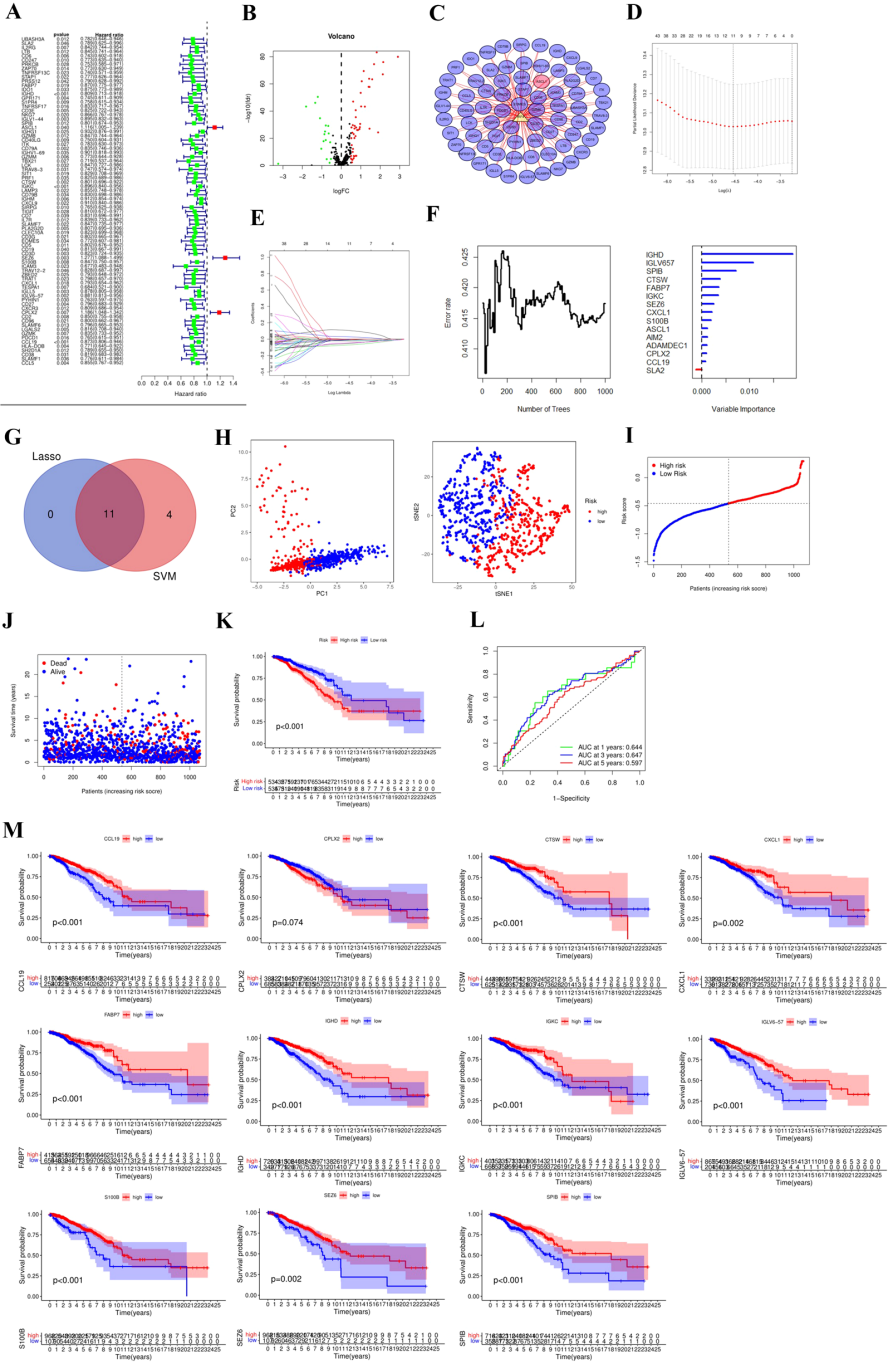
Establishment of prognostic risk model in TCGA. **A** Identification of prognosis-related genes via univariate cox analysis. **B** Differentially expressed TFs was shown in a volcano. **C** The regulatory network between TFs and ARGs. **D** Cross-validation for tuning the parameter selection in the LASSO regression. **E** 11 ARGs was identified via Lasso regression. **F** Screening the feature genes via SVM and obtaining 14 hub genes of importance. **G** Venn gram between Lasso and SVM. **H** PCA and TSNE plot for classification of patients based on the risk score. **I** Distribution of patients based on the risk score. **J** The survival status of patients (low-risk population: on the left side of the dotted line; high-risk population: on the right side of the dotted line). **K** Kaplan–Meier OS curves of patients between high and low-risk group. **L** ROC curves evaluated the predictive efficiency of the risk model

Univariate Cox analysis was first used to identify prognosis-related genes, of which 78 were closely related to prognosis (Fig. 3A, Table 2). On this basis, we identified differentially expressed TFs and constructed the TF-ARGs regulatory network (Fig. 3B, C). Based on the optimal λ value, 11 risk genes (Fig. 3D, E) were selected from 78 candidate genes through multivariate Cox regression analysis, namely, *FABP7*, *IGHD*, *SPIB*, *CTSW*, *IGKC*, *SEZ6*, *S100B*, *CXCL1*, *IGLV6-57*, *CPLX2* and *CCL19*. In order to further screen the feature risk genes, SVM was used to predict the feature genes. We identified 14 feature genes of greater than 0 importance (Fig. 3F), and then crossed them. Eventually, the core risk gene was obtained in Fig. 3G. The sum of the product of the gene expression and the corresponding regression coefficient was the prognostic risk score. Formula: Risk score = (−0.0522* *FABP7* exp.) + (−0.0703* *IGHD* exp.) + (−0.0200* *SPIB* exp.) + (−2.4926* *CTSW* exp.) + (−0.0071* *IGKC* exp.) + (0.1079* *SEZ6* exp.) + (−0.0037* *S100B* exp.) + (−0.0039* *CXCL1* exp.) + (−0.0108* *IGLV6-57* exp.) + (0.0300* *CPLX2* exp.) + (−0.0449* *CCL19* exp.). Based on the median prognostic risk score, 1076 patients in the training group were divided into high-and low-risk subgroups. The PCA and TSNE analyses suggested that patients in the high-and low-risk groups could be well divided into two independent clusters (Fig. 3H). Patients in the high-risk group had significantly more deaths and shorter survival times than those in the low-risk group (Fig. 3IJ). Survival analysis also showed that high-risk patients had a worse prognosis (*P* < 0.001) (Fig. 3K). The AUC value of the ROC curve was used to evaluate the predictive performance of the model. We found that the AUC was 0.644 in one year, 0.647 in three years and 0.597 in five years (Fig. 3L). The survival analyses of key genes of the model were as follows in Fig. 3M.

**Table 2:** The prognosis-related candidate genes via univariate Cox analysis in TCGA

Id	HR	HR.95L	HR.95H	*P* value
UBASH3A	0.78	0.64	0.94	0.01
SLA2	0.78	0.62	0.99	0.04
IL2RG	0.84	0.74	0.95	0.01
LTB	0.84	0.74	0.96	0.01
CD6	0.74	0.60	0.91	0.01
CD247	0.77	0.63	0.94	0.01
PRKCB	0.75	0.58	0.97	0.02
ZAP70	0.77	0.62	0.94	0.01
TNFRSF13C	0.73	0.57	0.95	0.02
STAP1	0.77	0.62	0.96	0.02
PRSS12	0.78	0.62	0.99	0.04
FABP7	0.87	0.77	0.97	0.01
IDO1	0.87	0.77	0.98	0.03
IGHD	0.80	0.71	0.91	0.00
GPR171	0.74	0.61	0.90	0.00
S1PR4	0.75	0.61	0.93	0.00
TNFRSF17	0.83	0.71	0.96	0.01
CD3E	0.82	0.72	0.94	0.00
NKG7	0.86	0.76	0.97	0.02
IGLV1-44	0.89	0.83	0.96	0.00
SPIB	0.80	0.67	0.95	0.01
ASCL1	1.11	1.00	1.23	0.03
IGHG1	0.93	0.87	0.99	0.02
GZMB	0.85	0.74	0.96	0.01
CD40LG	0.75	0.60	0.93	0.01
ITK	0.78	0.63	0.97	0.03
CD79A	0.84	0.75	0.94	0.00
IGHV1-69	0.90	0.82	0.99	0.03
GZMM	0.77	0.64	0.93	0.01
TBX21	0.72	0.54	0.96	0.03
LCK	0.85	0.73	0.99	0.03
TRAV8-3	0.75	0.57	0.97	0.03
SIT1	0.83	0.71	0.97	0.02
PRF1	0.82	0.69	0.99	0.03
CTSW	0.80	0.70	0.92	0.00
IGKC	0.90	0.84	0.96	0.00
LAMP3	0.86	0.75	0.98	0.02
CD79B	0.83	0.70	0.99	0.03
IGHM	0.91	0.85	0.97	0.01
CXCL9	0.91	0.84	0.99	0.02
SIRPG	0.77	0.62	0.94	0.01
TIGIT	0.81	0.67	0.98	0.03
CD7	0.83	0.70	0.99	0.04
IL7R	0.84	0.73	0.96	0.01
SLAMF7	0.85	0.74	0.98	0.02
PLA2G2D	0.81	0.69	0.94	0.00
CLEC10A	0.82	0.70	0.97	0.02
CD3G	0.80	0.67	0.97	0.02
EOMES	0.77	0.61	0.98	0.03
CD5	0.80	0.68	0.95	0.01
CD19	0.81	0.67	0.99	0.04
CD3D	0.82	0.72	0.93	0.00
SEZ6	1.28	1.09	1.50	0.00
S100B	0.85	0.75	0.96	0.01
ICAM3	0.68	0.48	0.95	0.02
TRAV12-2	0.83	0.69	1.00	0.05
ZBED2	0.79	0.65	0.97	0.03
TRAT1	0.80	0.66	0.97	0.02
CXCL1	0.79	0.65	0.96	0.02
TESPA1	0.68	0.52	0.90	0.01
IGLL5	0.88	0.81	0.96	0.00
IGLV6-57	0.88	0.81	0.96	0.00
PYHIN1	0.76	0.60	0.97	0.03
CD27	0.80	0.68	0.93	0.00
CXCR3	0.81	0.69	0.95	0.01
CPLX2	1.19	1.05	1.34	0.01
CD2	0.85	0.75	0.96	0.01
CD96	0.80	0.66	0.97	0.02
SLAMF6	0.80	0.66	0.95	0.01
LGALS2	0.82	0.71	0.94	0.00
GZMK	0.83	0.73	0.95	0.01
PDCD1	0.76	0.62	0.95	0.02
CCL19	0.87	0.81	0.95	0.00
HLA-DOB	0.77	0.64	0.92	0.00
SH2D1A	0.79	0.66	0.95	0.01
CD38	0.82	0.68	0.98	0.03
SLAMF1	0.78	0.61	0.98	0.04
CCL5	0.85	0.77	0.95	0.00

### External validation of the risk model

**Fig. 4:**
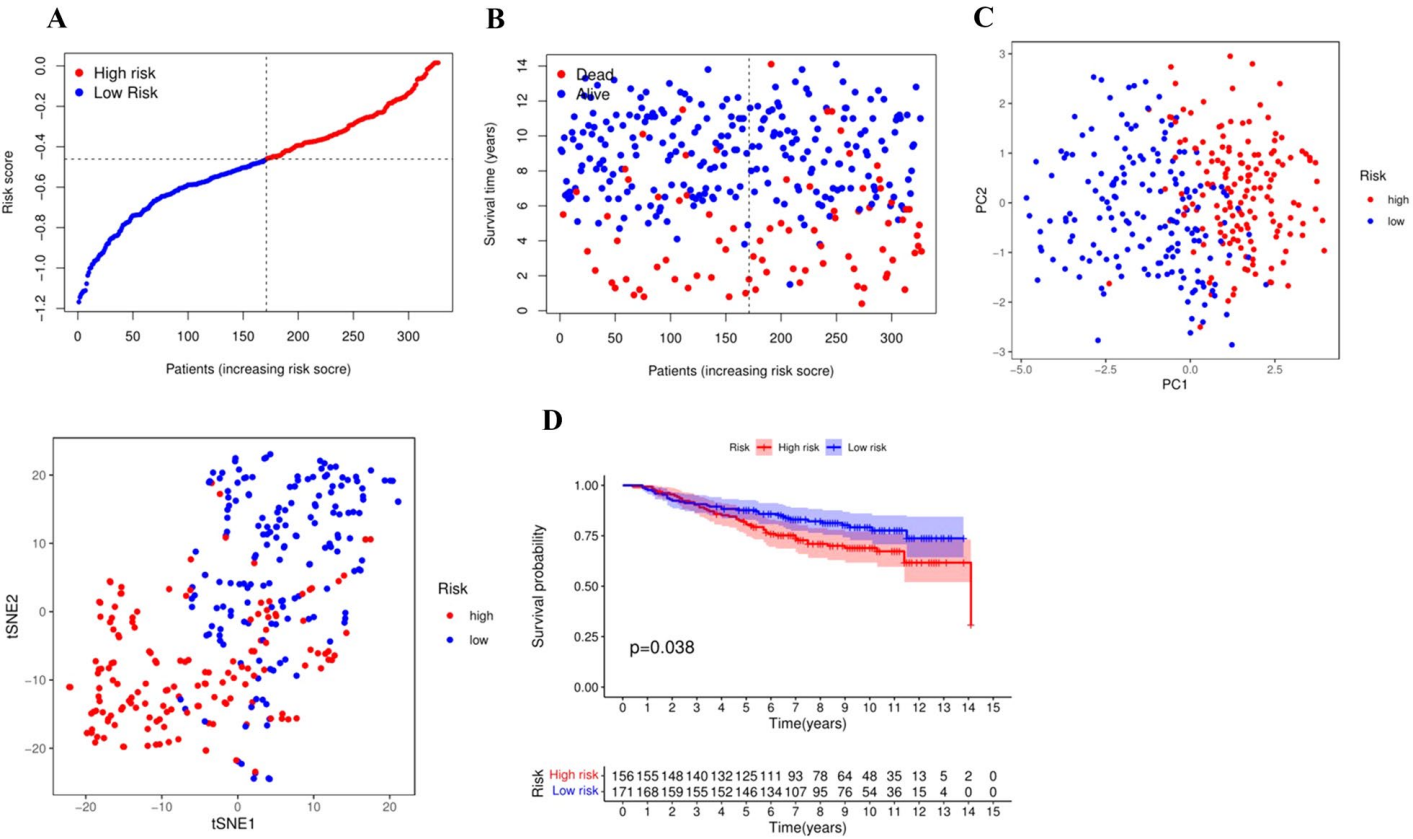
External validation of the risk model. **A** Distribution of patients in the GEO cohort based on the median risk score in the TCGA cohort. **B** The survival status of patients in GEO cohort. **C** PCA and TSNE classification of patients in GEO cohort. **D** Kaplan–Meier survival curve for prognostic signature in training set

GSE158309 was utilized to validate the model as a testing set. The calculation of the prognostic risk score and the high–low risk grouping of patients remained the same, and patients in the testing set were also divided into the high–low risk group (Fig. 4A). Similar to the training set, the death numbers in the high-risk group was significantly higher than that in the low-risk group (Fig. 4B, C). The Kaplan–Meier survival curve analysis also showed a significant reduction in OS (*P* = 0.038) (Fig. 4D), which suggested that the model had the same prognostic assessment ability for patients in an external independent cohort.

### Independent prognostic analysis of risk model

**Fig. 5:**
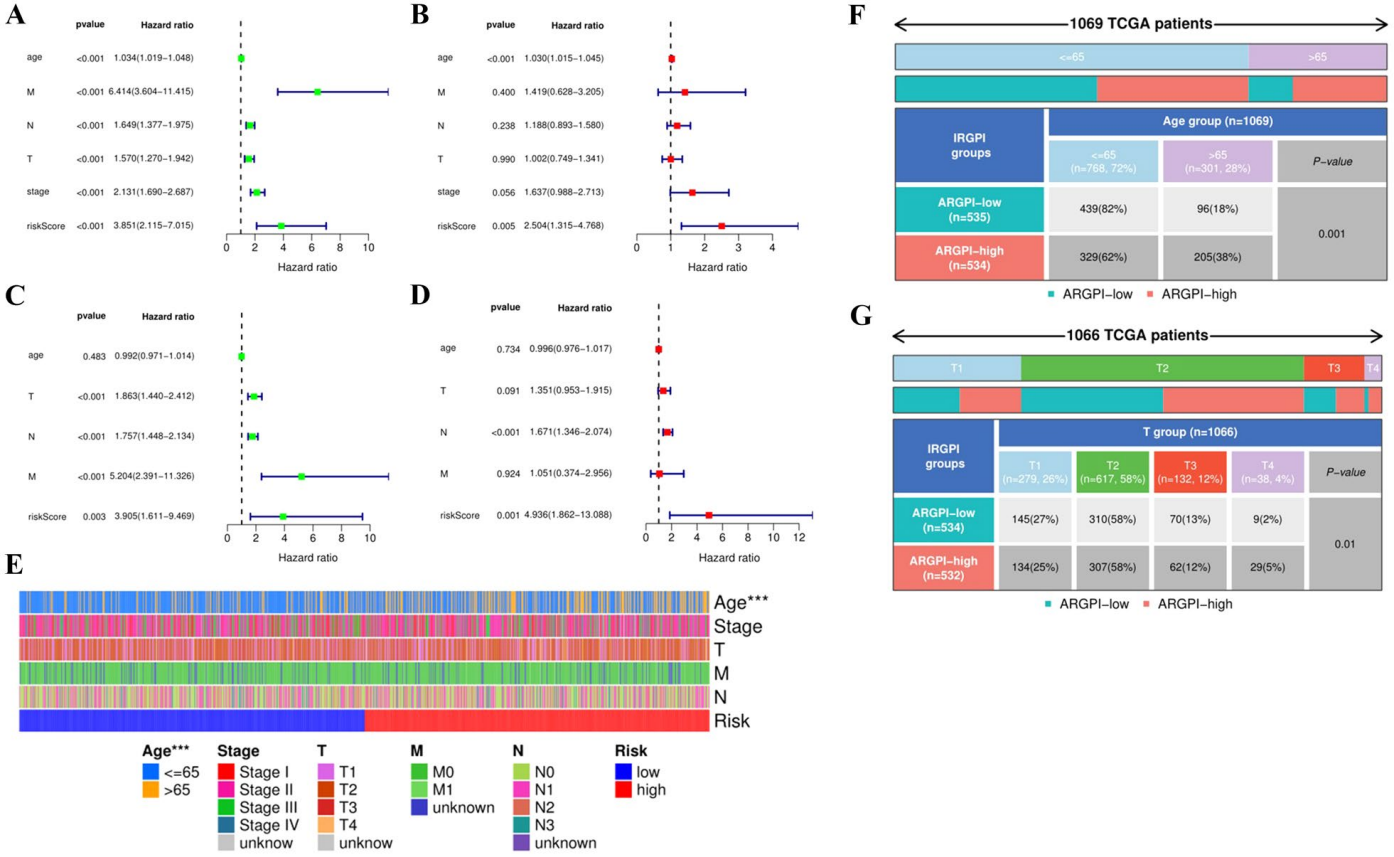
Results of the univariate and multivariate cox regression for the risk score. **A** Univariate cox analysis for the TCGA cohort. **B** Multivariate cox analysis for the TCGA cohort. **C** Univariate cox analysis for the GEO cohort. **D** Multivariate cox analysis for the GEO cohort. **E** The connections between clinicopathologic features and the risk groups. **F** The connections and difference between age groups and the risk groups. **G** The connections and difference between T stage (1 to 4) groups and the risk groups

Univariate and multivariate Cox analyses were performed to assess whether the risk score was an independent risk factor for predicting prognosis. The results of univariate Cox analysis showed that risk score, age, stage and pathological grade were independent risk factors, and multivariate Cox analysis determined the risk score and age as reliable independent risk factors (Fig. 5A, B). Same analysis on the testing set was also performed, and the results also confirmed that risk score was an independent risk factor for the development of BC (Fig. 5C, D, Table 3). Furthermore, we performed a correlation analysis of the clinical characteristics of patients in the high- and low-risk groups, and heatmaps indicted that age was generally higher in the high-risk group than in the low-risk group (*P* = 0.001), there were also significant differences between T stage (*P* = 0.032), while other clinical features not (Fig. 5E–G).

**Table 3:** Clinical parameters and risk score via univariate and multivariate Cox regression analyses

Variables	Univariate analysis				Multivariate analysis			
	HR	HR.95L	HR	*P*	HR	HR.95L	HR.95H	*P*
TCGA								
Age	1.03	1.02	1.05	3.41e−06	1.03	1.01	1.05	9.62e−05
M	6.41	3.60	11.41	2.64e−10	1.42	0.63	3.21	0.40
N	1.65	1.38	1.98	5.35e−08	1.19	0.89	1.58	0.24
T	1.57	1.27	1.94	3.07e−05	1.00	0.75	1.34	0.99
Stage	2.13	1.69	2.69	1.57e−10	1.64	0.99	2.71	0.06
Risk score	3.85	2.11	7.01	1.04e−05	2.50	1.32	4.77	0.01
GEO								
Age	0.99	0.97	1.01	0.48	1.00	0.98	1.02	0.73
T	1.86	1.44	2.41	2.28e−06	1.35	0.95	1.91	0.09
N	1.78	1.45	2.13	1.22e−08	1.67	1.35	2.07	3.17e−06
M	5.20	2.39	11.33	3.22e−05	1.05	0.37	2.96	0.92
Risk score	3.91	1.61	9.47	0.00	4.94	1.86	13.09	0.00

### Construction of a nomogram

**Fig. 6:**
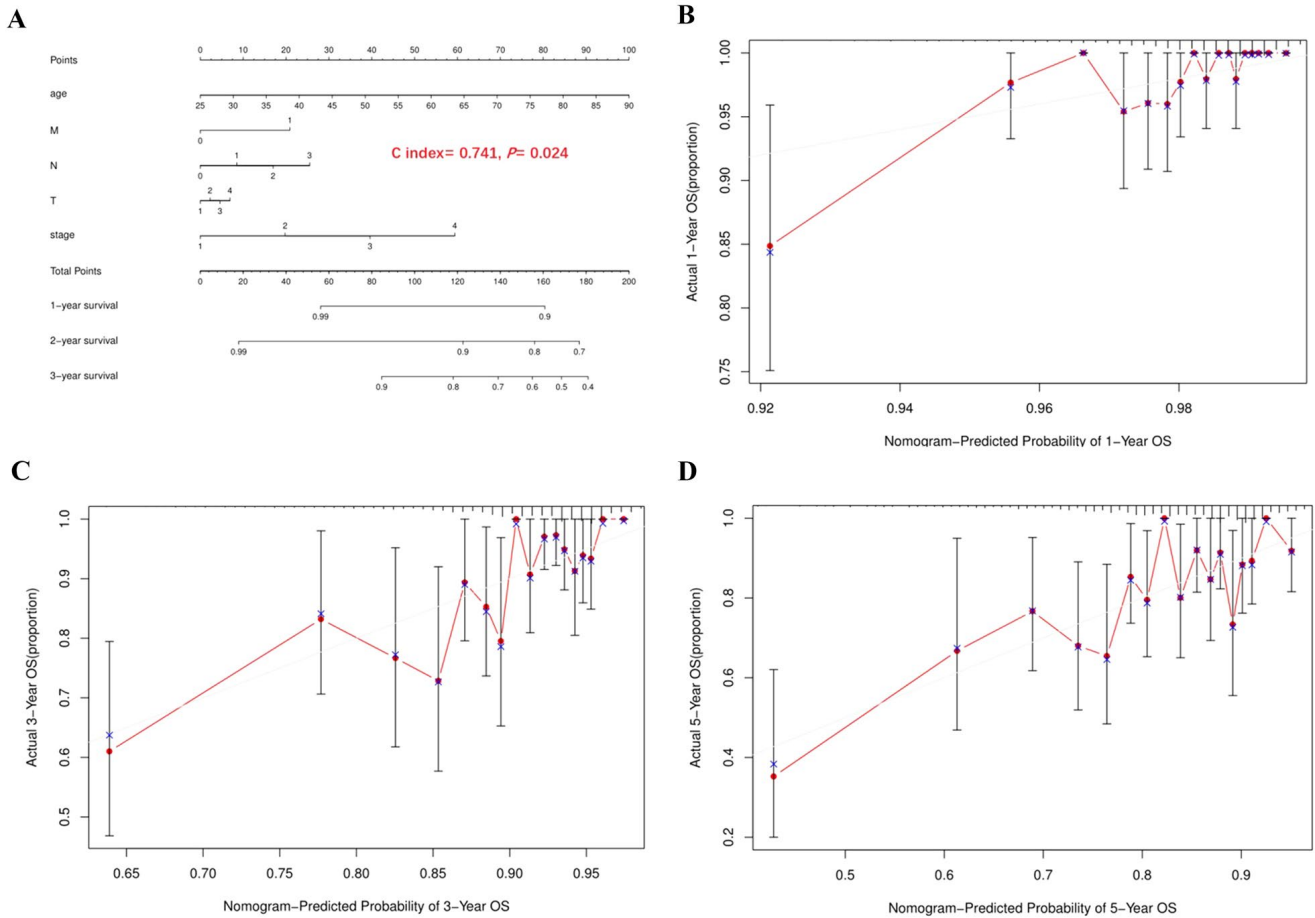
The construction of a nomogram to predict the OS of the patients. **A** A nomogram combined clinical features with risk score. **B** Calibration curve for predicting BC patients’ OS at 1 year. **C** Calibration curve for predicting BC patients’ OS at 3 years. **D** Calibration curve for predicting BC patients’ OS at 5 years

Nomogram is a powerful tool of visualizing predictive models, making them more operable and practical. Therefore, in order to apply our risk model to clinical practice, we combined risk score with clinical characteristics including age, tumor grade, T stage, N stage and N stage, subsequently a nomogram was constructed to predict 1, 2, and 3-year survival in BC patients (Fig. 6A). Besides, the calibration curve showed that the nomogram could effectively predict the actual survival prognosis (C index = 0.74090825, *P* = 0.02372858 Fig. 6B–D).

### Gene set enrichment analysis (GSEA)

GSEA was conducted to investigate the signal pathways associated with the characteristics associated with aging. GO results showed that DNA replication dependent nucleosome organization, mRNA 5 splice site recognition, regulation of skeletal muscle adaptation, U4 snRNP and U6 snRNA binding were enriched in high-risk group, while adaptive immune response, myeloid leukocyte mediated immunity, positive regulation of locomotion, taxis, and side of membrane were enriched in low-risk group (FDR < 0.05). KEGG pathway analysis indicated cardiac muscle contraction and drug metabolism other enzymes were enriched in high-risk group, while cell adhesion molecules cams, chemokine signaling pathway, cytokine–cytokine receptor interaction, JAK–STAT signaling pathway and pathways in cancer were enriched in low-risk group (FDR < 0.05) (Additional file 1: Fig. S1A–D). In an effort to further explore the differences of function and pathway among subgroups classified by risk model, we screened the DEGs with the criteria of FDR < 0.05 and | log2FC | ≥ 1 via the “limma” R package. A total of 171 DEGs were identified between the low-risk and high-risk groups in the TCGA cohort. In the high-risk group, 19 genes were up-regulated and 152 genes were down-regulated (Additional file 4: Table S2). In-depth GO analysis showed that the differentially expressed genes in high-and low-risk groups were mainly enriched in immune-related biological processes, such as humoral immune response, leukocyte migration, regulation of inflammatory response and adaptive immune response (Additional file 1: Fig. S1E). In terms of KEGG, the results of the analysis also suggested that the differentially expressed genes in the high-risk and low-risk groups were mainly enriched in the immune-related pathway, such as Th1 and Th2 cell differentiation, Th17 cell differentiation, T cell receptor signaling pathway and Natural killer cell mediated cytotoxicity (Additional file 1: Fig. S1F). Next, we conducted the PPI network to construct and identify the hub genes (high confidence = 0.7), where the first 10 genes were *CD8A*, *PTPRC*, *CD2*, *CD19*, *CCR7*, *CXCR3*, *LCK*, *CXCL9*, *IL7R* and *PRF1*, which occupied an essential role in occurrence and development of BC (Additional file 1: Fig. S1G, H).

### Composition of immune infiltrating cells and immune-related functions between high- and low-risk groups

**Fig. 7:**
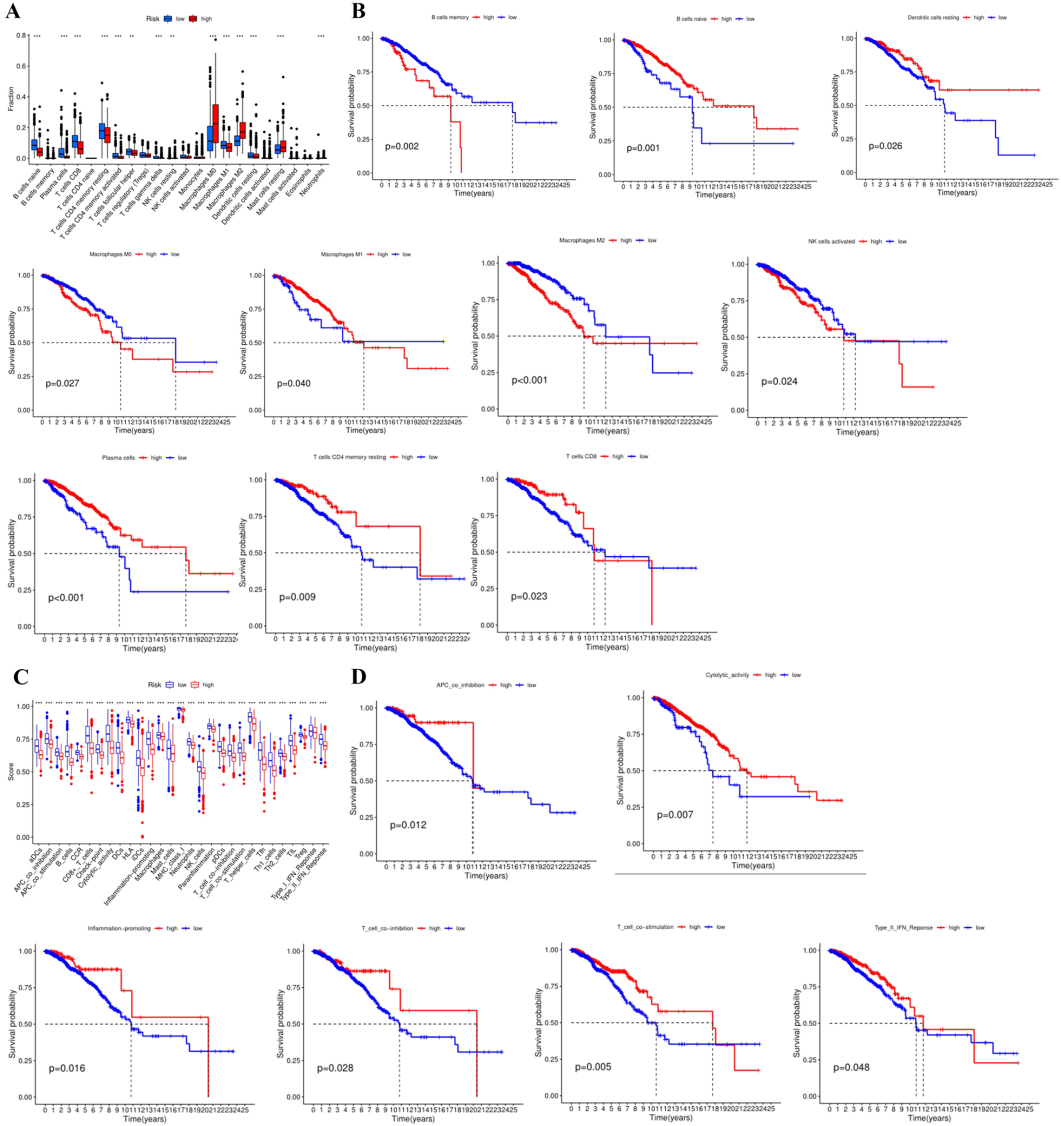
The enrichment of immune cells and immune-related function between different risk groups. **A** The abundance of 24 immune cells are displayed in a boxplot. **B** Kaplan–Meier OS curves for the patients between high and low-level immune cells. **C** The abundance of 13 immune-related functions are displayed in a boxplot. **D** Kaplan–Meier OS curves for the patients between high and low-level immune-related functions

In order to better understand the prognostic risk model and the immunobiological characteristics of tumor immune microenvironment, we evaluated the tumor immune infiltrating cells enriched in the high-and low-risk groups. The CIBERSORT algorithm calculated the relative proportion of immune cells in the immune microenvironment of BC patients. We found that B cells naïve, Plasma cells, T cells CD8, T cells CD4 memory resting, T cells CD4 memory activated, T cells follicular helper, T cells gamma delta, NK cells resting, Macrophages M1 and Dendritic cells resting were enriched in low-risk group patients, while Macrophages M0, M2, Mast cells resting and Neutrophils were enriched in high-risk group patients (Fig. 7A). In addition, we performed a survival analysis of the high and low expression of immune cells, indicating that the prognosis of patients with high enrichment of B cells memory (*P* = 0.002), Macrophages M0 (*P* = 0.027), Macrophages M2 (*P* < 0.001), NK cells activated (*P* = 0.024) was significantly worse than that of patients with low enrichment, while high enrichment of B cells memory (*P* = 0.002), Macrophages M0 (*P* = 0.027), Macrophages M2 (*P* < 0.001), NK cells activated (*P* = 0.024) had a better prognosis (Fig. 7B). Moreover, we calculated the abundance and distribution of enriched immune-related function in patients with high and low-risk groups to further assess the relationship between risk score and immune status, the finding was similar with the infiltrating immune cells where the enrichment score of immune-related function in the low-risk group was higher than that in the high-risk group, including APC co-inhibition, APC co-stimulation, Inflammation-promoting, Cytolytic-activity, T cell co-inhibition, T cell co-stimulation, Type I IFN response, Type II IFN response, which further indicated that the low-risk group was in a high anti-tumor immune state and exhibited anti-tumor effect (Fig. 7C). This was further confirmed by survival analysis of immune-related functions (Fig. 7D).

### Mutation analysis

**Fig. 8:**
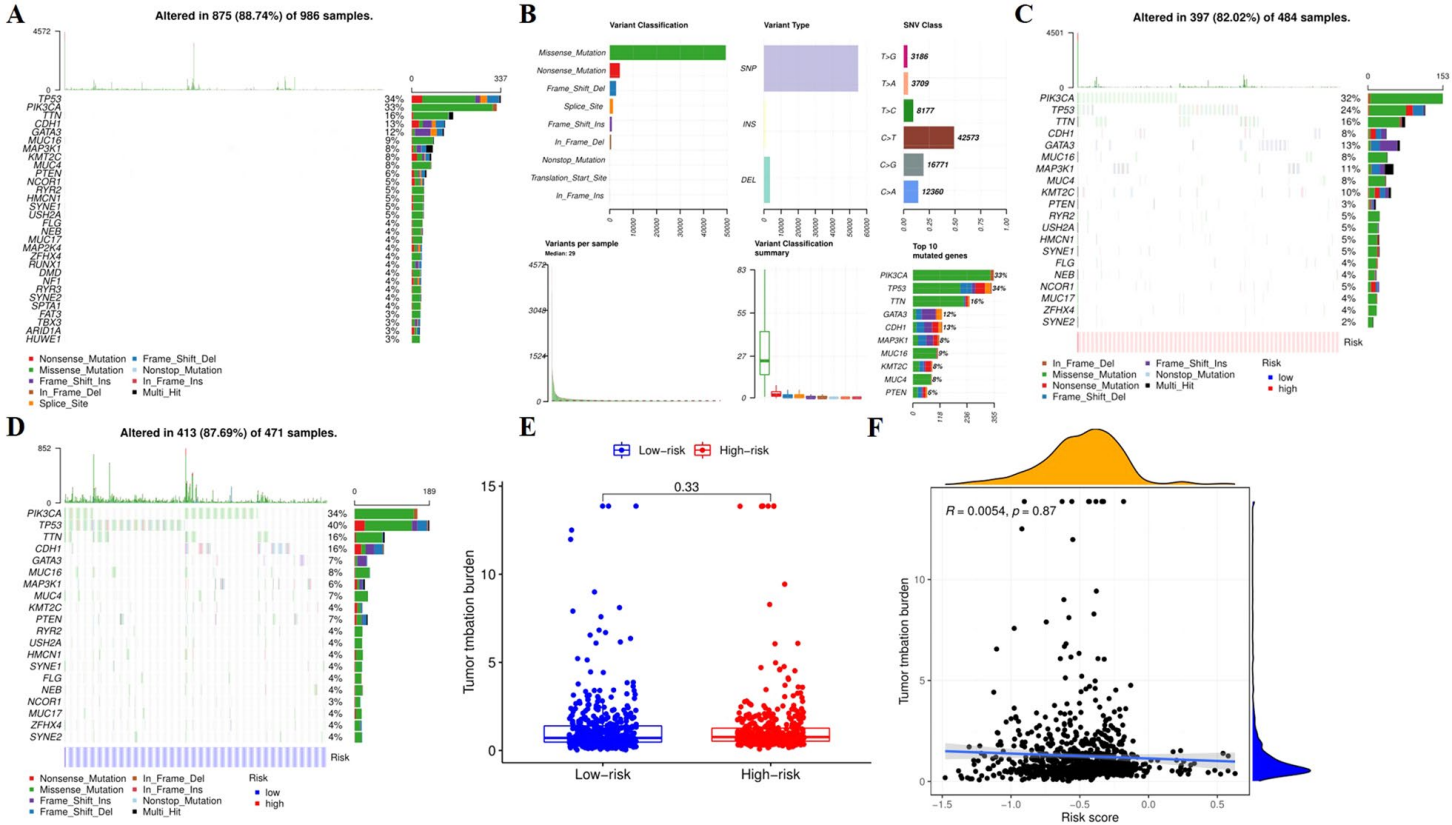
Mutations spectrum of BC patients. **A** Waterfall plot representing the mutant landscape of the top 20 most frequently mutated genes in all samples. **B** Summary plot showing the types of gene variants. **C** Waterfall plot representing the mutant landscape of the top 20 most frequently mutated genes in the low-risk group. **D** Tumor mutational burden between high- and low-risk groups. E. The relationship between tumor mutational burden and the risk score

To explore the mechanism of development of BC, we studied the mutation profiles of ARGs. Based on the “maftools” package, we analyzed and visualized the mutations in each sample and found that 875 out of 986 samples had mutations, with TP53 (34%) being the most frequent mutation in the first three genes, followed by PIK3CA (33%) and TTN (16%). The most common type of mutation is missense, while the most common type of SNV is C > T (Fig. 8A, B). We also investigated the gene mutations in the high-and low-risk groups. The results indicated that the gene with the highest frequency of mutation was *PIK3CA*, *TP53*, and *TTN* in high-risk patients, the most frequent mutations were *TP53*, *PIK3CA* and *TTN* in the low-risk group, but the most common mutations in both groups were missense mutations (Fig. 8C, D). We also calculated the TMB, which was defined as the number of mutations detected per million bases, based on the type and frequency of gene mutations in each patient, the total number of errors in somatic gene coding, base substitution, gene insertion, or deletion. At present, TMB is the newest marker to evaluate the therapeutic effect of PD-1 antibody, being severely significant to evaluate the TMB score of high and low-risk group for BC patients. Nevertheless, through correlation analysis, we found that there was no significant negative correlation between TMB and risk score, and there was no significant difference in TMB score between high and low-risk groups (Fig. 8E, F).

### Tumor microenvironment

**Fig. 9:**
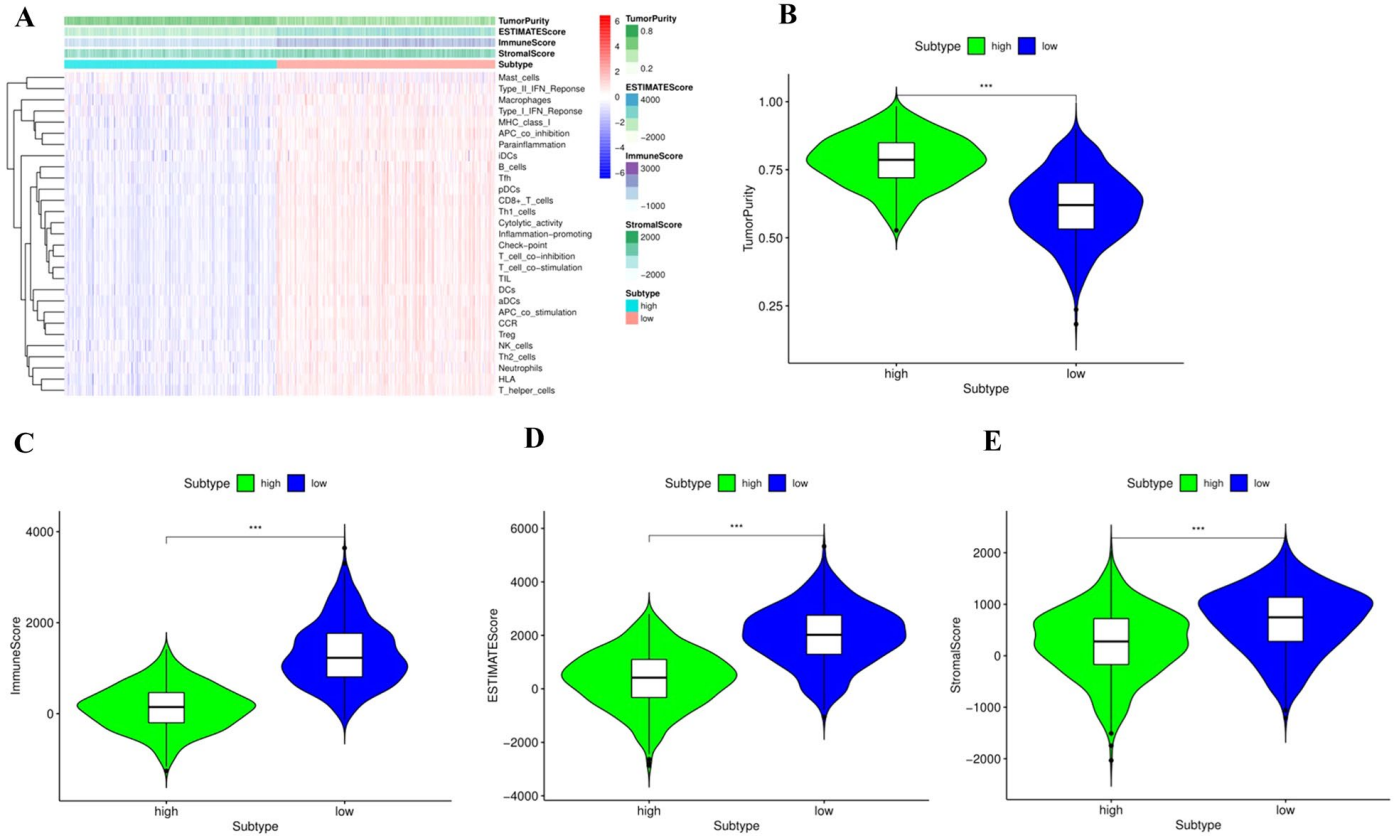
The immune landscape of BC patients between high- and low-risk groups. **A** The immune status of patients between high- and low-risk groups. **B** The tumor purity of patients between high- and low-risk groups. **C** The immune score of patients between high- and low-risk groups. **D** The ESTIMATE score of patients between high- and low-risk groups. **E** The stromal score of patients between high- and low-risk groups

Tumor tissue includes not only tumor cells, but also many cells related to tumor microenvironment, such as stromal cells, immune cells and so on. Different types of immune cells play different roles in anti-tumor and tumor immune escape, and tumor growth, invasion and metastasis. Stromal cells are also associated with tumor growth and disease progression, so in addition to CIBERSORT analysis of immunologic invasion, we also evaluated the proportion and abundance of immune cells, stromal cells and tumor cells in each patient’s tumor tissue using ESTIMATE and ssGSEA. According to the abundance of immune cells, stromal cells and tumor cells in tumor tissue, we calculated the immune score, stromal score, tumor purity and ESTIMATE score of each patient (Fig. 9A). More importantly, we compared the differences between the high-risk group and the low-risk group. The results showed that the tumor purity score in the high-risk group was significantly higher than that in the low-risk group (*P* < 0.001), while the matrix score, the immune score and the ESTIMATE score were significantly lower, which was consistent with our previous analysis (*P* < 0.001) (Fig. 9B–E).

### Tumor Immune Dysfunction and Exclusion (TIDE)

**Fig. 10:**
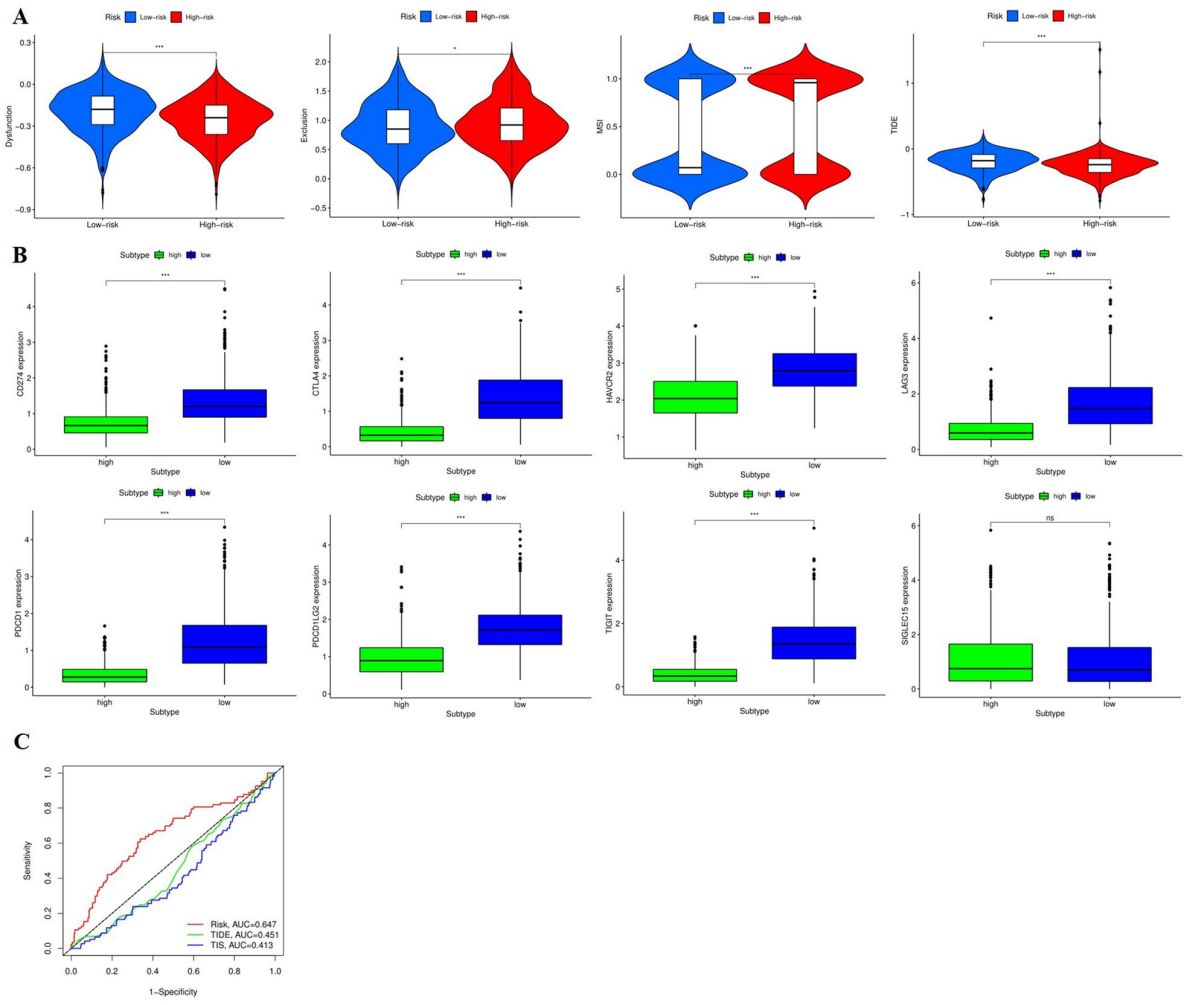
Correlation of risk scores with Tumor Immune Dysfunction and Exclusion and 8 immune checkpoints. **A** Violin plots visualizing correlation of TIDE scores with the high- and low-groups. **B** Boxplots showing comparison of the expression of immune checkpoints between the high- and low-groups. **C** ROC curves demonstrating the comparison between the risk score and the TIDE score, and TIS score

TIDE is an algorithm that utilizes gene expression markers to assess two different mechanisms of tumor immune escape, including the dysfunction of tumor infiltrating cytotoxic T lymphocyte and the rejection of CTL by immunosuppressive factors which can well predict the response of a single sample or a subtype to predicted immune checkpoint inhibitor. Dysfunction score and exclusive score were the two main components of TIDE score, in which dysfunction score was used to assess immune dissonance genes, and exclusive score was used to assess immune rejection genes. Therefore, TIDE algorithm was used to calculate different scores and predict the potential response of each patient to immunotherapy. Results showed that low-risk patients had significantly lower exclusion scores than high-risk patients, while dysfunction and TIDE scores were significantly higher than high-risk patients, which suggested that the high-risk group may be more effective on ICB. In addition, we calculated the MSI score. MSI refers to any change in microsatellite length caused by insertion or deletion of repeat units, and the appearance of a new microsatellite allele in a tumor compared with normal tissue, which can not only predict the prognosis of patients, can also reflect the direction of follow-up immunotherapy. Our results suggested that MSI scores in high-risk group are significantly higher, indicating that the high-risk group may also have a better response to immunotherapy (Fig. 10A). Mounting studies have shown that immunotherapy is becoming a new hope for cancer treatment, and immune checkpoint proteins play an important role in immune response. Therefore, we compared the expression levels of common checkpoint proteins in high-risk and low-risk groups. The results showed that the expression of *CD274*, *CTLA4*, *HAVCR2*, *LAG3*, *PDCD1*, *PDCD1LG2*, *TIGIT* in the low-risk group was significantly higher than that in the high-risk group (*P* < 0.001) (Fig. 10B). We also explored the risk model with the TIDE and TIS scores, and found that the risk score model had better predictive sensitivity and specificity (Fig. 10C).

### The key genes in risk model

To further elucidate the relationship between risk genes and immune cells and mutations, we conducted an in-depth study in the TIMER database. The SCNA module provides a comparison of tumor invasion levels between tumors with different copy number changes for a given gene, which was characterized by GISTIC 2.0 with deep deletion, arm-level deletion, diploid/normal, arm-level gain and high amplification. Wilcoxon rank sum test was performed to compare the infiltration level of each SCNA class with the normal level in TIMER database. It was found that different types of mutations in the risk model genes could alter tumor infiltrating immune cells such as CD4^+^ T cells, CD8^+^ T cells and so on (Additional file 2: Fig. S2A). Meanwhile, we explored and identified that the correlation between the expression level of risk-related genes and the proportion of infiltrating cells existed (Additional file 2: Fig. S2B, C). First, we identified the expression of the risk model genes in all TCGA tumors. It was found that the expression of *CPLX2*, *CTSW*, *CXCL1*, *FABP7*, *S100B*, *SEZ6* and *SPIB* were all differentially expressed in BC (Additional file 2: Fig. S2D), further, we examined the mRNA levels of the core genes in normal breast tissue and BC tissue. We found that the expressions of *CCL1*, *FABP7*, *S100B*, *CTSW*, and *SEZ6* significantly decreased in the tumor tissue, the expression of *CPLX2* and *SPIB* increased obviously in BC (Additional file 2: Fig. S2E). The expression of *ICKC* also increased in GSE38959, *IGLV6-57* increased in GSE70905, nevertheless, *IGHD* and *IGKC* decreased in BC tissues in GSE17907 (Additional file 2: Fig. S2F). Besides, the protein expression of these genes was investigated in HPA, which was consistent with the mRNA expression (Additional file 2: Fig. S2G). Finally, we also analyzed the proportion of all patients with TCGA who had mutations in risk genes, with the highest rates of mutations in the genes *SEZ6*, *SPIB* and *CTSW*, consistent with the entire genome, and the most common types of mutations being missense mutations, the SNV is C > T (Additional file 2: Fig. S2H, I).

### Chemosensitivity of BC between groups

**Fig. 11:**
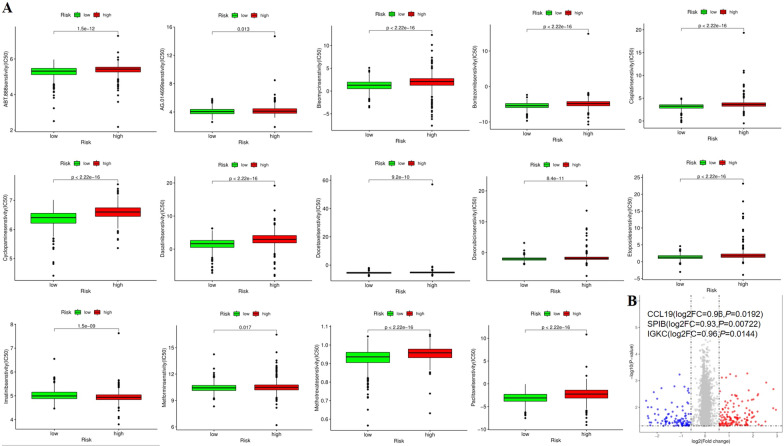
The relationship between risk groups and chemosensitivity. **A** There was significant difference for chemosensitivity between high- and low-risk groups. **B** The expression of *CCL19*, *SPIB and IGKC* differed between after chemotherapy

In addition to the immunotherapies explored above, we also studied the sensitivity of chemotherapeutic drugs and targeted drugs to the treatment of patients in high-and low-risk groups, the IC50 of chemotherapeutic drugs such as cisplatin, paclitaxel and doxorubicin was significantly lower in the low-risk group than in the high-risk group, suggesting that chemotherapeutic drugs may have better sensitivity and efficacy in the low-risk group (Fig. 11A). We also investigated the effects of conventional chemotherapy on risk genes, and using the DREAM database, we found significant changes in the expression levels of many genes, including *CCL19* (FC = 0.96, *P* = 0.0192), *SPIB* (FC = 0.93, *P* = 0.00722) and *IGKC* (FC = 0.96, *P* = 0.0144) in BC patients treated with drugs (Fig. 11B).

### Protein–protein interactions (PPI) of hub genes at the GeneMANIA

GeneMANIA is used to predict the functional similarity of central genes. We obtained 20 similar genes from the hub gene. The central gene is located in the inner circle, while the prediction gene is located in the outer circle. Their function is focused on Chemokine receptor binding, which is closely related to the previously studied chemokines and the onset of aging. ERK1 and ERK2 regulatory pathways and the proliferation and apoptosis of DC cells are also the major functional pathways of age-related proteins (Additional file 3: Fig. S3).

### Validation of 11‐gene prognostic signature

**Fig. 12:**
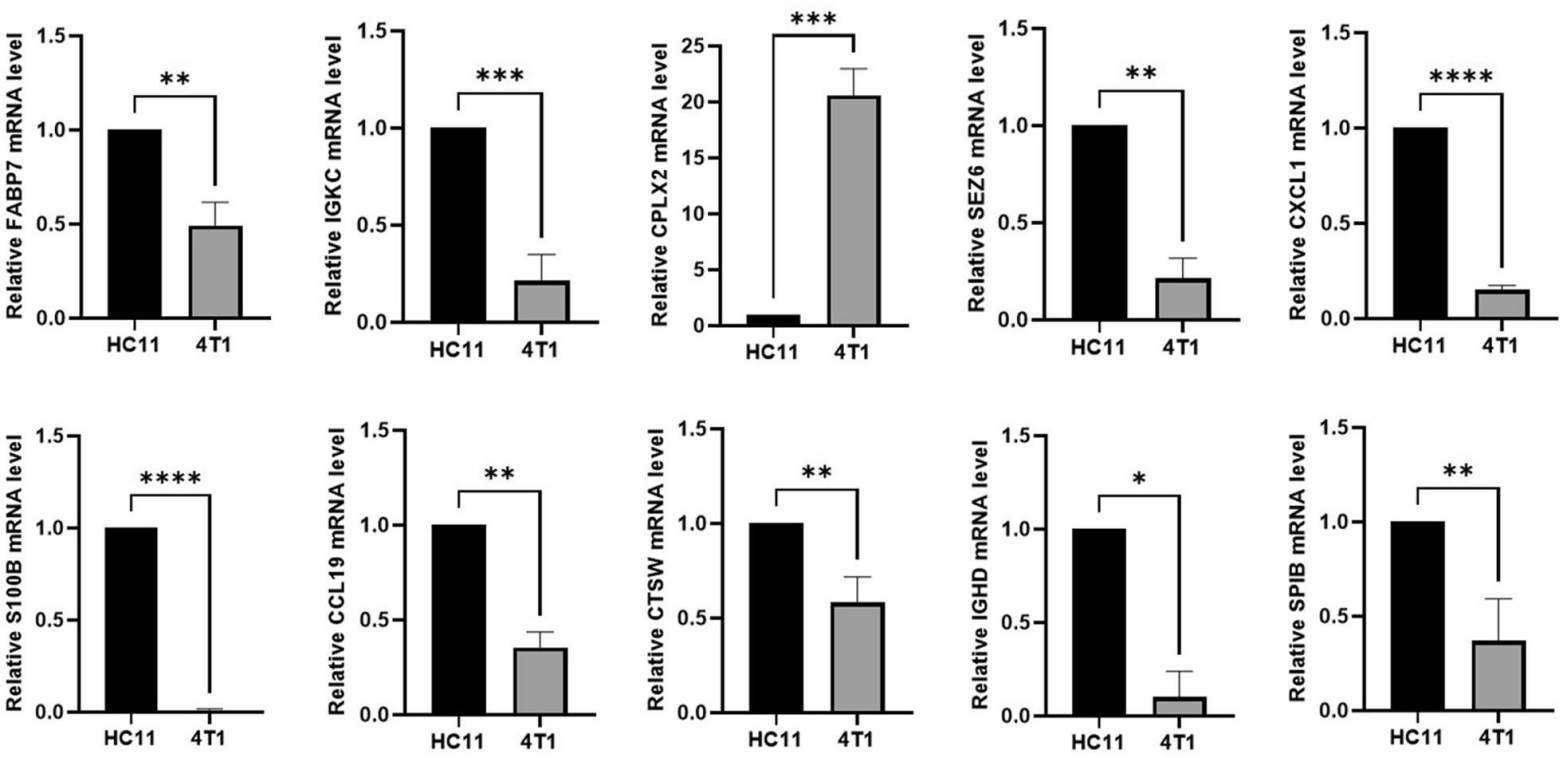
Validation of the expression of the risk genes via RT–PCR

To further verify the accuracy of the 11‐gene prognostic signature, we detected the expression levels of in BC cell line and normal breast cell line by RT–PCR. The experimental results revealed that the protective prognostic factors in BC patients were significantly down-regulated, whereas the dangerous prognostic factors were up-regulated except *SEZ6* (Fig. 12).

## Discussion

BC is one of the most common malignant tumors threatening women’s health with an increasing incidence rate year by year. The latest global cancer data for 2020 released by the World Health Organization’s International Agency for Research on cancer, showed that BC has surpassed lung cancer to become the malignant with highest incidence and mortality worldwide. Moreover, the incidence of BC peaks in older People (> 50 years) [14], while the incidence of BC gradually increases in younger women (< 35 years), which is generally more aggressive. Previous studies have shown that the incidence of BC increases with age and that the occurrence of cancer is related to the accumulation of replicative senescence in normal human cells and p21-induced senescence in tumor cells, suggesting that aging is involved in the development of BC [15]. Complicatedly, the aging-related pathway is contradictory in the process of tumorigenesis. In the early stage of tumorigenesis, aging is a protective mechanism against tumor transformation, while in advanced periods, which promotes tumor growth by altering the microenvironment [16]. Therefore, aging is one of the mechanisms by which normal cells avoid tumorigenesis, and is also an important part of the survival advantage of cancer itself. Taken together, there is a pressing necessity for exploring the expression of ARGs in BC and understanding the role of aging process in the development of BC.

**Fig. 13:**
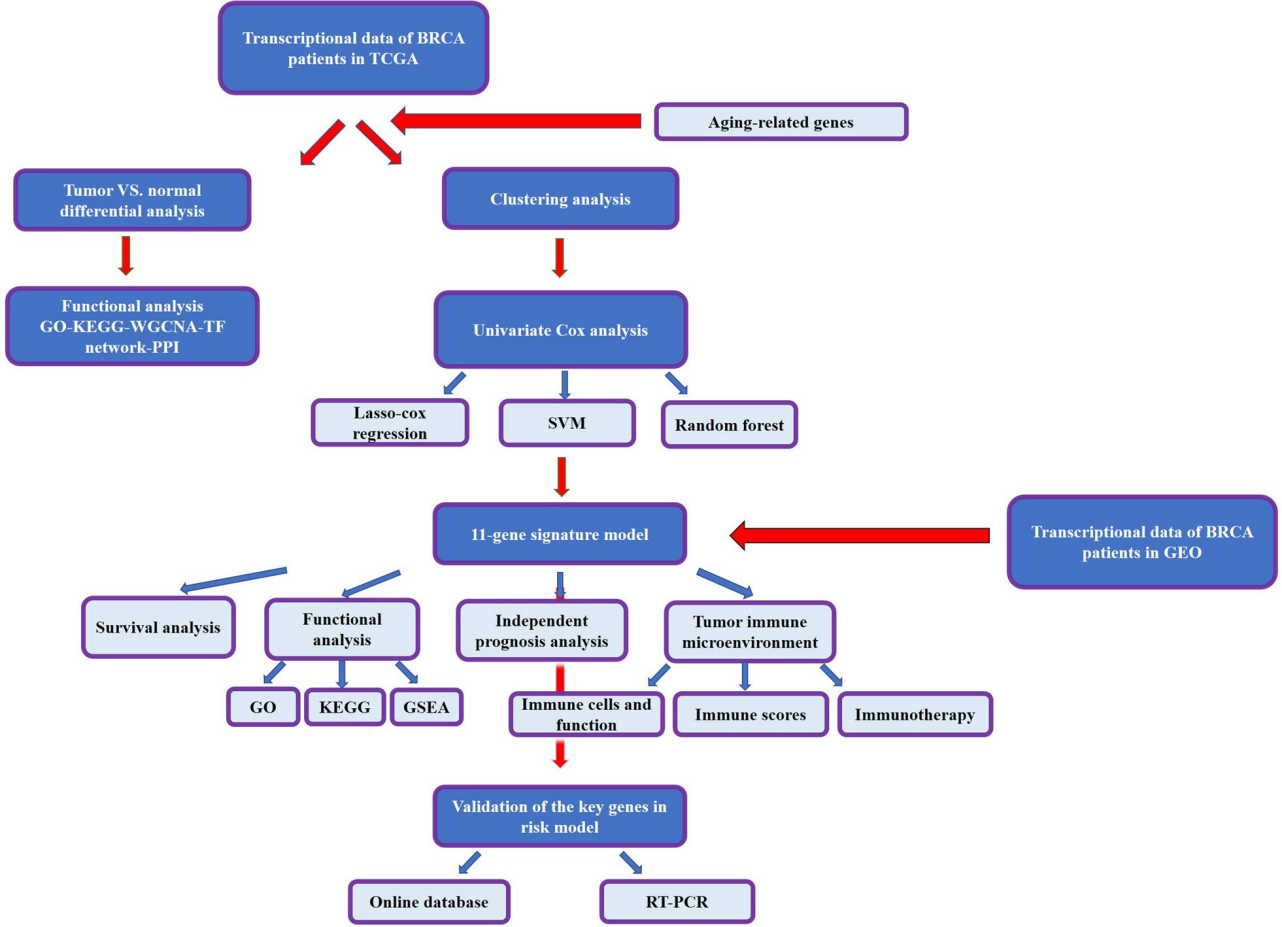
The flowchart of the current study

In this study, we first performed a comprehensive analysis to identify differentially expressed ARGs and core prognostic genes in BC, and then constructed a risk model consisting of 11 ARGs, evaluated and validated for its ability to predict the survival outcome of BC patients. The flowchart is shown in Fig. 13.

In this risk model, *FABP7*, *IGHD*, *SPIB*, *CTSW*, *IGKC*, *S100B*, *CXCL1*, *IGLV6-57*, *CCL19* were protective factors, while *SEZ6* and *CPLX2* were risk factors. *FABP7* is a member of the fatty acid binding protein family and is thought to facilitate the transport of fatty acids in various organelles and regulate their metabolism and other physiological activities [17, 18]. Recent studies showed that *FABP7* was significantly involved in the pathogenesis and progression of many types of cancer and could be used as a promising tumor marker [18]. The expression of *FABP7* in BC tissue was found to be significantly lower than that in normal tissue samples, which could enhance chemosensitivity by regulating cell cycle. And the high expression of *FABP7* predicted better chemotherapy response and longer relapse-free survival [19]. When it referred to *IGHD*, it is located in the variable region of coding H in the chromosome 14(14q32.33), which is an important component of the antigen binding loop of immunoglobulins. Studies have found increased levels of *IGHD* mRNA in AML patients, and *IGHD* was an independent risk factor for OS in AML patients [20]. Besides, *IGHD* has also been studied in BC, being a protective factor for BC recurrence and functioning as tumor suppressor [21]. *SPIB* is a member of the ETS transcription factor family and is abnormally activated at all stages of tumorigenesis [22]. Xu found that *SPIB* played an important role in the occurrence and development of tumors. Overexpression of *SPIB* inhibited cell proliferation and induces apoptosis in children with acute lymphocyte leukemia [23]. Not only that, *SPIB* had anti-apoptotic effect in diffuse large B-cell Lymphoma via PI3K–AKT pathway [24], which was associated with the poor prognosis and could be identified as a prognostic indicator in HCC [25]. A prognostic model for BC bone metastases identified SPIB as a protective factor for BC bone metastases, and its high expression predicted better outcomes in BC patients, consistent with our findings [26]. *CTSW* is a novel human cysteine protease expressed in CD8 T cells and NK cells [27], and plays an important role in cytotoxicity mediated by NK cells and CD8 T cells [28, 29]. The study conducted by Chen found that *CTSW* was positively correlated with the prognosis of patients in endometrial cancer, and the expression level of *CTSW* was positively correlated with tumor infiltrating immunity, suggesting that *CTSW* might inhibit tumor progression by regulating tumor immune microenvironment [30].

Consistent with our study, multiple BC-related surveys have identified that *IGKC*, Immunoglobulin Kappa C, is an independent prognostic factor in BC patients, and high expression is significantly associated with good DFS and OS [31].

The S100 calcium-binding protein family is an inflammatory molecule that contributes to the formation of an inflammatory tumor microenvironment [32]. There are more than 20 genes in the S100 family, some of which are considered tumor markers. *S100B* is exactly a member of this family [33]. To date, *S100B* has been proven to be a cancer-promoting factor, whose overexpression can promote the proliferation, invasion and metastasis of lung cancer cells, exhibiting significant negative correlation with the prognosis of patients [34, 35]. However, the role of *S100B* in BC was quite contrary. High expression of *S100B* was associated with better prognosis in BC. Taken together, the research illustrated that *S100B* could inhibit the migration of ER negative BC, functioning as a predictor of BC metastasis [36].

C-X-C motif chemokine ligand 1 (*CXCL1*) is the most abundant chemokine secreted by tumor associated macrophages, which is located on chromosome 4. Elevated levels of *CXCL1* in CRC were associated with tumor size, progression, depth of invasion, and patient survival [37, 38]. And in BC, *CXCL1* could promote the migration and invasion of BC through NF-κB/SOX4 signaling pathway, which was contrary to our research [39].

An article on prognostic markers of colorectal cancer identified *IGLV6-57* as a core risk gene in the risk model. The higher the expression of *IGLV6-57*, the worse the patients’ prognosis [40]. However, no study has investigated an association between *IGLV6-57* and BC. Therefore, further analysis is needed to explore the relationship between these genes and pathways and BC in order to guide the diagnosis and treatment of BC.

The chemokine CC Ligand 19 (*CCL19*), also known as macrophage inflammatory protein 3-β (MIP-3b), mediates various cellular behaviors by binding to CCR7 [41]. It was found that *CCL19* expression significantly decreased in CRC, which was closely related to tumor proliferation. Further investigation revealed that *CCL19* could inhibit CRC angiogenesis by promoting miR-206 and inhibiting the Met/ERK/Elk-1/HIF-1α/VEGF-A pathway, which might be a new therapeutic option for anti-vascular therapy of CRC [42]. In BC, however, *CCL19* appeared to play a role as a cancer-promoting gene. Overexpression of *CCL19* induced invasion and metastasis of BC cells [43].

*SEZ6*, an active regulated mRNA transcript, is indispensable for the development of dendrites and synapses, and involving in the development of chronic hyperalgesia and neuroinflammation following nerve injury [44]. Patients with high expression of *SEZ6* had better prognosis [45]. Nevertheless, the involvement of *SEZ6* in the development and progression of BC has not been reported.

*CPLX2* is a member of the complex protein/synaptophysin family involved in the regulation of synaptogenesis and the release of neurotransmitters in the presynaptic terminals of the brain [46]. Komatsu et al. identified that *CPLX2*, a potential biomarker for high-level human lung neuroendocrine tumors (L-NETs), was significantly down-regulated in L-NETs, and its high expression was associated with lymphatic infiltration, pathological stage and survival [47]. A recent study has also shown that *CPLX2* was up-regulated in glioblastoma multiforme tissue compared to normal brain tissue, serving as an effective marker [48].

Above all, these results conclusively supported our findings that these ARGs influenced tumor progression and patients’ survival. We then combined clinical features and risk scores to demonstrate the potential of risk score as an independent risk factor and to construct a nomogram for predicting survival in BC patients. Besides, we explored the relationship between risk score and clinical parameters, and the results clearly indicated that there was significant difference of risk score between different clinical groups, such as age and T stage, which verified that the risk signature was correlated with clinical parameters. Interestingly, the subsequently functional enrichment analysis of differentially expressed ARGs in the high-risk and low-risk groups revealed that multiple immune pathways were enriched in the low-risk groups, suggesting that ARGs might be closely related to immunity. Especially, in terms of Th1 and Th2 cell differentiation, it has been found to be closely related to the immune microenvironment and immunotherapy of BC. Taken together, our results have reconfirmed the fundamental role of Th1–Th2 status for BC patients. Pathologically, aging could lead to metabolic disorders, decreased immune response and malnutrition, and might induce many chronic diseases, including cancer [49, 50]. Previous studies have shown that the immune response in tumors is usually triggered by aging, and the infiltration of immune cells in the tumor microenvironment contributes to tumor growth [51]. Aging is associated with impaired immune function and the accumulation of chronic inflammatory microenvironment, which may promote tumor formation and progression [52]. Anti-tumor immune impairment is a typical example of immune aging [53]. For these reasons, we studied differences in the abundance of immune cells and immune function in the tumor microenvironment of patients in the high-and low-risk groups. As we previously determined, there was a significant enrichment of anti-tumor immune cells in the low-risk group, while in the high-risk group, the proportion of cancer-promoting immune cells such as M2 macrophages was higher. We even explored different immune scores in the high-and low-risk groups, which indicated that patients in low-risk group had higher immune score and lower tumor purity, and demonstrated that risk score was highly associated with tumor immune microenvironment.

Therefore, our study revealed a significant correlation between risk scores and immune status. Among all key genes, *CCL19* played an important role in the formation and maintenance of T cell regions in lymphoid organs [54]. Overexpression of *CCL19* exhibited an important role in anti-tumor activity and tumor clearance [55]. In terms of *CPLX2*, it could inhibit the B cell antibody secretion [56]. In addition, *CXCL1* was also an important component of the tumor microenvironment, which was significantly associated with the abundance of B cells, DC cells, CD8 + T cells, etc., regulating tumor microenvironment [57]. Our study also found that changes in the copy number variation (CNV) of most of the risk genes significantly altered the abundance of immune cells in the tumor immune microenvironment, which indicated that CNV of core genes might also be one of the important mechanisms of BC. Previous studies also demonstrated that CNV was closely related to the risk factors of BC. The incidence of BC was high in CNV carriers, and there was a causal relationship between CNV and the incidence of BC [58–61], owing to the occurrence of CNV at multiple alleles and the presence of more large copy number loss or amplification during DNA replication, leading to severer histological grade and the more malignant degree so as to recur and metastasize. Based on above findings, we also explored the CNV situation in the high-and low-risk groups. The mutation spectrum in the two groups was not completely same, but further studies are needed to clarify the differences.

Above studies have provided strong evidence that the ARGs were strongly associated with the immune microenvironment of BC, and also suggested that the immune response in tumors was often triggered by aging. However, little is known about the association between immunotherapies and aging in BC [51]. At present, BC immunotherapy program is in full swing. Tumor immunotherapy involves reactivating the body’s anti-tumor immune response to kill tumor cells, including vaccines, chimeric antigen receptor T cell therapy, and immunocheckpoint inhibitors, of which immunocheckpoint inhibitors are of the most concern.

We first explored the expression of immune checkpoint genes such as *SIGLEC15*, *TIGIT*, *CD274*, *HAVCR2*, *PDCD1*, *CTLA4*, *LAG3* and *PDCD1LG2* in high-and low-risk groups. Immune checkpoint molecules expressed on immune cells will inhibit the function of immune cells, so that the body could not produce an effective anti-tumor immune response, contributing to tumor immune escape. Our results demonstrated that checkpoint molecule expression was significantly higher in the low-risk group than in the high-risk group. Further research into the response of high and low-risk groups to immune checkpoint blockade (ICB) treatment found that the high-risk group was better treated with ICB, which suggested that we could further identify BC immunotherapy beneficiaries through our risk model for adequate treatment.

Except for immunotherapy, chemotherapy remains mainstream treatment in BC and our results also indicated that patients in low-risk group benefited more from multiple chemotherapy drugs, such as Cisplatin and Paclitaxel, providing precise personalized treatment for different cancer patients.

Ultimately, we conducted an in-depth exploration of the model genes, and the expression profile analysis proved that these model genes were differentially expressed in BC. At the same time, the pan-cancer analysis results also found that they also had certain differences in various tumors, which showed that the model genes might be related to the occurrence and development of tumors to a certain extent, providing a certain clue for future in-depth exploration. More than that, in our model, most genes are strongly associated with TME in cancer. For example, CCL19, CTSW, S100B, etc., are significantly positively correlated with macrophages, CD8+ T cells, etc., offering some clues for subsequent studies.

However, there are still some limitations in our study. First, our experimental data are based on the results of these retrospective analyses of TCGA and GEO, which may be biased. Therefore, we need to use prospective, multicenter datasets for further validation; Secondly, we have only studied the risk model and the potential relationship between core genes and the tumor immune microenvironment, but there is no definite regulating pathway, so we still need to explore the specific mechanism. Thirdly, we should carry out functional experiments on these aging genes to elucidate their role in the development of BC. Eventually, there are many molecular types of BC, and the pathological mechanisms of different molecular types are not the same, and the treatment options are also different. Therefore, further classification of specific subtypes will be more helpful to provide individualized treatment for patients.

In summary, in our study, we developed a robust prognostic risk model with 11 ARGs that assessed and demonstrated risk scores as independent prognostic indicators. Meanwhile, we also demonstrated a strong association between risk score and core genes and the tumor immune microenvironment, producing highly sensitive biomarkers for BC patients and providing new clues for individualized immunotherapy.

## Conclusions

In conclusion, we developed and validated a robust prognosis-related risk model based on 11 ARGs, which could effectively predict the survival of BC patients and provide a promising biomarker in the diagnosis of BC and a new direction into tumor immune microenvironment and immunotherapy.

## Supplementary Information

**Additional file 1: Figure S1.** Functional analyses between high and low-risk groups. **A** Gene set enrichment analysis in high-risk group referred as the c5.go.v7.4.symbols.gmt gene set. **B** Gene set enrichment analysis in low-risk group referred as the c5.go.v7.4.symbols.gmt gene set. **C** Gene set enrichment analysis in high-risk group referred as the c2.cp.kegg.v7.4.symbols.gmt gene set. **D** Gene set enrichment analysis in low-risk group referred as the c2.cp.kegg.v7.4.symbols.gmt gene set. **E** Bubble plot for Go enrichment based on the DEGs between the risk groups in TCGA cohort. **F** Bubble plot for KEGG enrichment based on the DEGs between the risk groups in TCGA cohort. **G** PPI network showing the interactions of the DEGs. **H** The obtained first 10 hub genes from the network.

**Additional file 2: Figure S2.** The relationship between key genes in the risk model and immune microenvironment. **A** Different types of SCNA of the key genes contributed to various enrichment in immune cells. **B** Correlation analyses between the expression level of the key genes and the immune cells. **C** Lollipop plots showing the correlation between immune cells and the expression of the key genes. **D** The expression of the key genes between normal and tumor tissue in various cancers. **E** The expression of the key genes between normal and tumor tissue in breast cancer in TCGA cohort. **F** The expression of the key genes between normal and tumor tissue in breast cancer in GEO cohort. **G** The protein expression of the key genes between normal and tumor tissue in breast cancer in HPA database. **H** The waterfall of the mutation landscape of the risk genes. **I** The summary plot showing the types of the gene mutation.

**Additional file 3: Figure S3.** PPI network to demonstrate the interactions of the risk genes at the GeneMANIA.

**Additional file 4: Table S1.** Aging-related genes downloaded from the Human Aging Genomic Resources 3. **Table S2.** Differentially expressed genes between high-risk and low-risk group patients in TCGA.

## Data Availability

All data about TCGA dataset (https://portal.gdc.cancer.gov), GEO dataset (https://www.ncbi.nlm.nih.gov/geo/), GeneMANIA (http://genemania.org/), GEPIA (http://gepia.cancer-pku.cn/index.html) and HPA (https://www.proteinatlas.org/) are publicly available.

## Abbreviations

BC: Breast cancer
ARGs: Aging-related genes
TCGA: The Cancer Genome Atlas
GEO: Gene expression omnibus
ROC: Receiver operating characteristic
RT-PCR: Real‐time polymerase chain reaction
HPA: Human Protein Atlas
TF: Transcription factors
OS: Overall survival
KM: Kaplan–Meier
GSEA: Gene Enrichment Analysis
TMB: Tumor mutation load
ssGSEA: Single-sample GSEA
DEG: Differentially expressed gene
TIDE: Tumor Immune Dysfunction and Exclusion
CNV: Copy number variation
ICB: Immune checkpoint blockade
