# Supplementary figures and images for: Establishment and validation of an aging-related risk signature associated with prognosis and tumor immune microenvironment in breast cancer
Source: Eur J Med Res. 2022 Dec 29;27:317. doi: 10.1186/s40001-022-00924-4 (PMC9798726; doi:10.1186/s40001-022-00924-4)

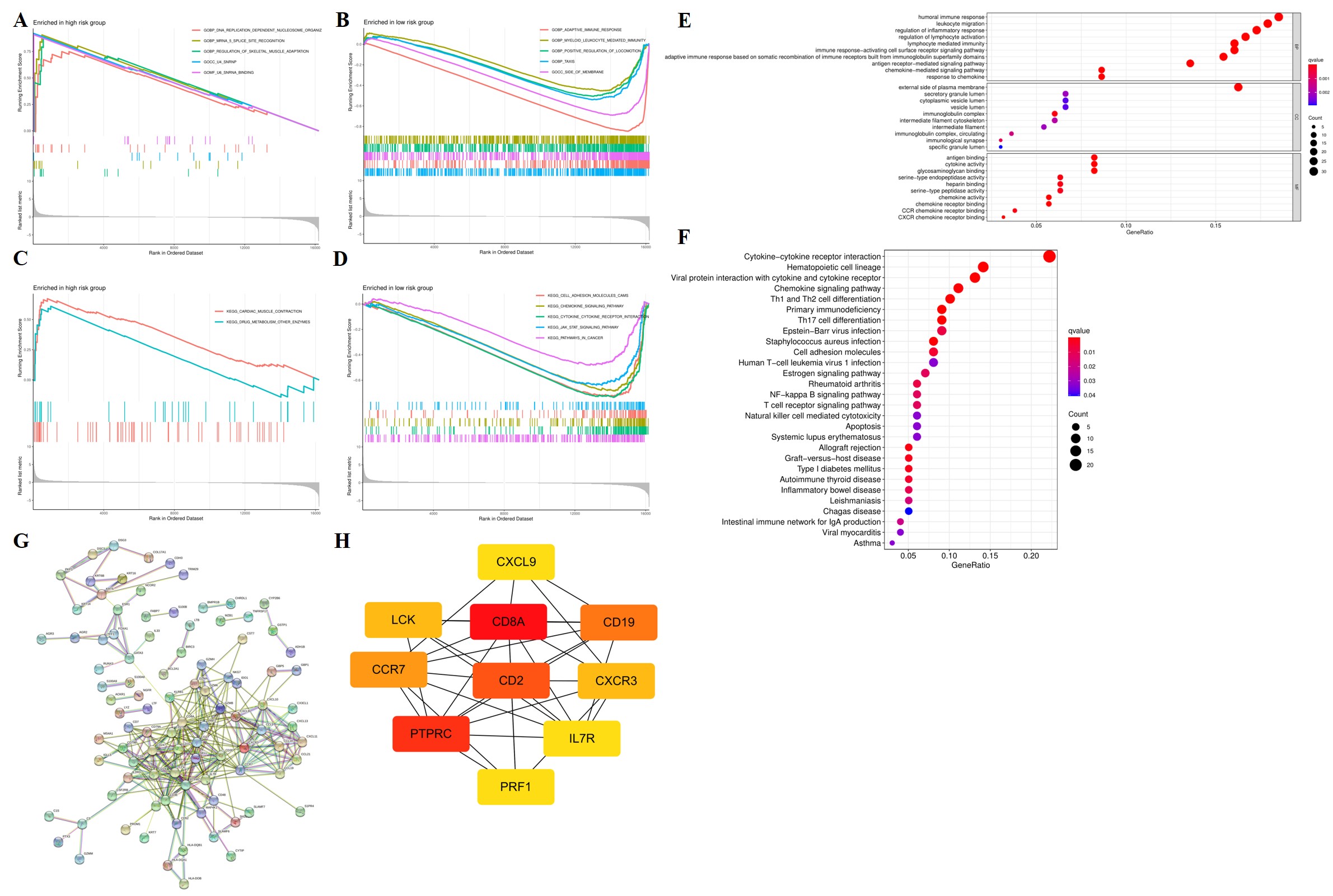

Supplement: Supplementary file 1 — Additional file 1: Figure S1. Functional analyses between high and low-risk groups. A Gene set enrichment analysis in high-risk group referred as the c5.go.v7.4.symbols.gmt gene set. B Gene set enrichment analysis in low-risk group referred as the c5.go.v7.4.symbols.gmt gene set. C Gene set enrichment analysis in high-risk group referred as the c2.cp.kegg.v7.4.symbols.gmt gene set. D Gene set enrichment analysis in low-risk group referred as the c2.cp.kegg.v7.4.symbols.gmt gene set. E Bubble plot for Go enrichment based on the DEGs between the risk groups in TCGA cohort. F Bubble plot for KEGG enrichment based on the DEGs between the risk groups in TCGA cohort. G PPI network showing the interactions of the DEGs. H The obtained first 10 hub genes from the network. [file 40001_2022_924_MOESM1_ESM.jpg]

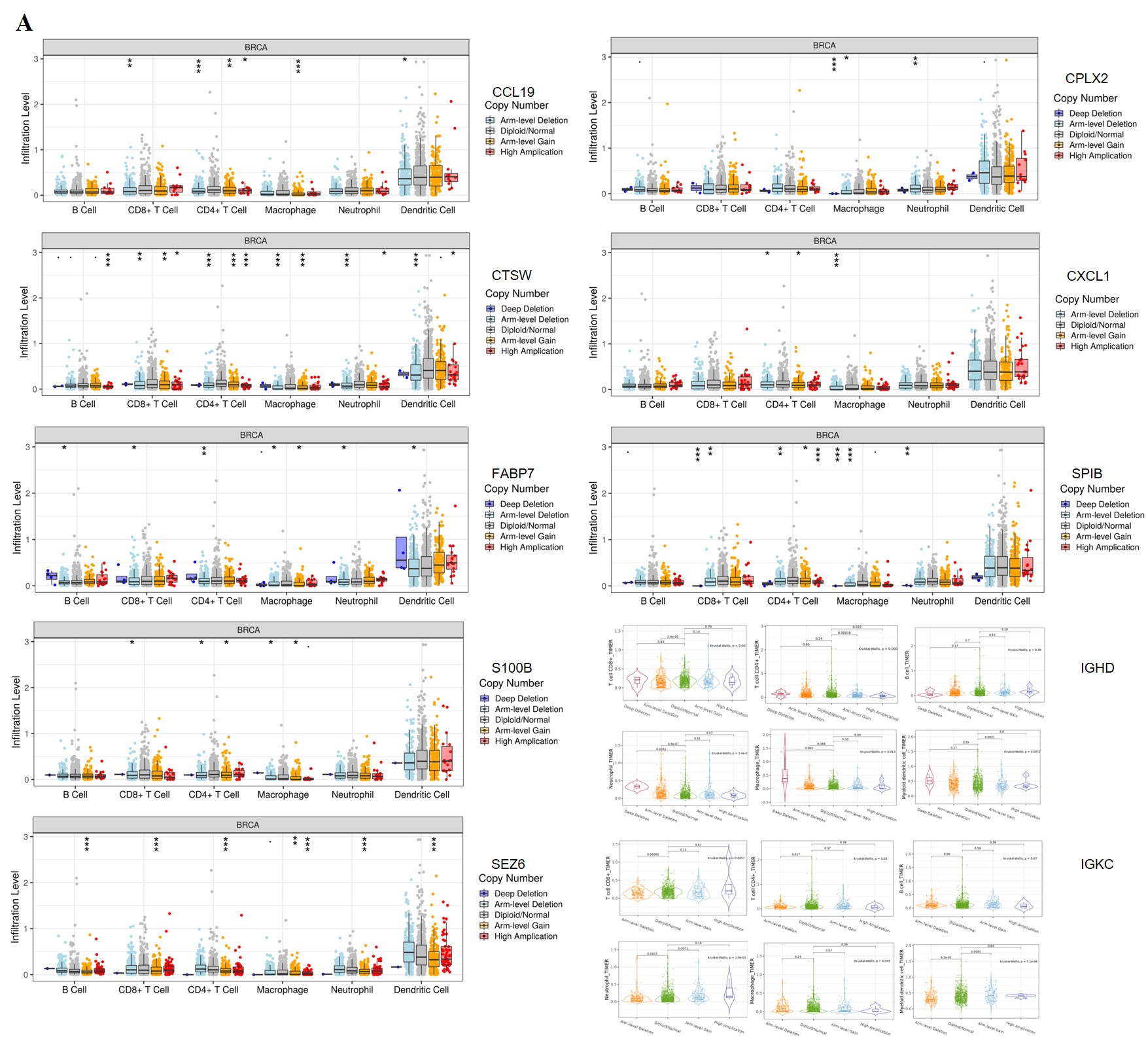

Supplement: Supplementary file 2 — Additional file 2: Figure S2. The relationship between key genes in the risk model and immune microenvironment. A Different types of SCNA of the key genes contributed to various enrichment in immune cells. B Correlation analyses between the expression level of the key genes and the immune cells. C Lollipop plots showing the correlation between immune cells and the expression of the key genes. D The expression of the key genes between normal and tumor tissue in various cancers. E The expression of the key genes between normal and tumor tissue in breast cancer in TCGA cohort. F The expression of the key genes between normal and tumor tissue in breast cancer in GEO cohort. G The protein expression of the key genes between normal and tumor tissue in breast cancer in HPA database. H The waterfall of the mutation landscape of the risk genes. I The summary plot showing the types of the gene mutation. [file 40001_2022_924_MOESM2_ESM.zip › Supplementary Figure 2A.jpg]

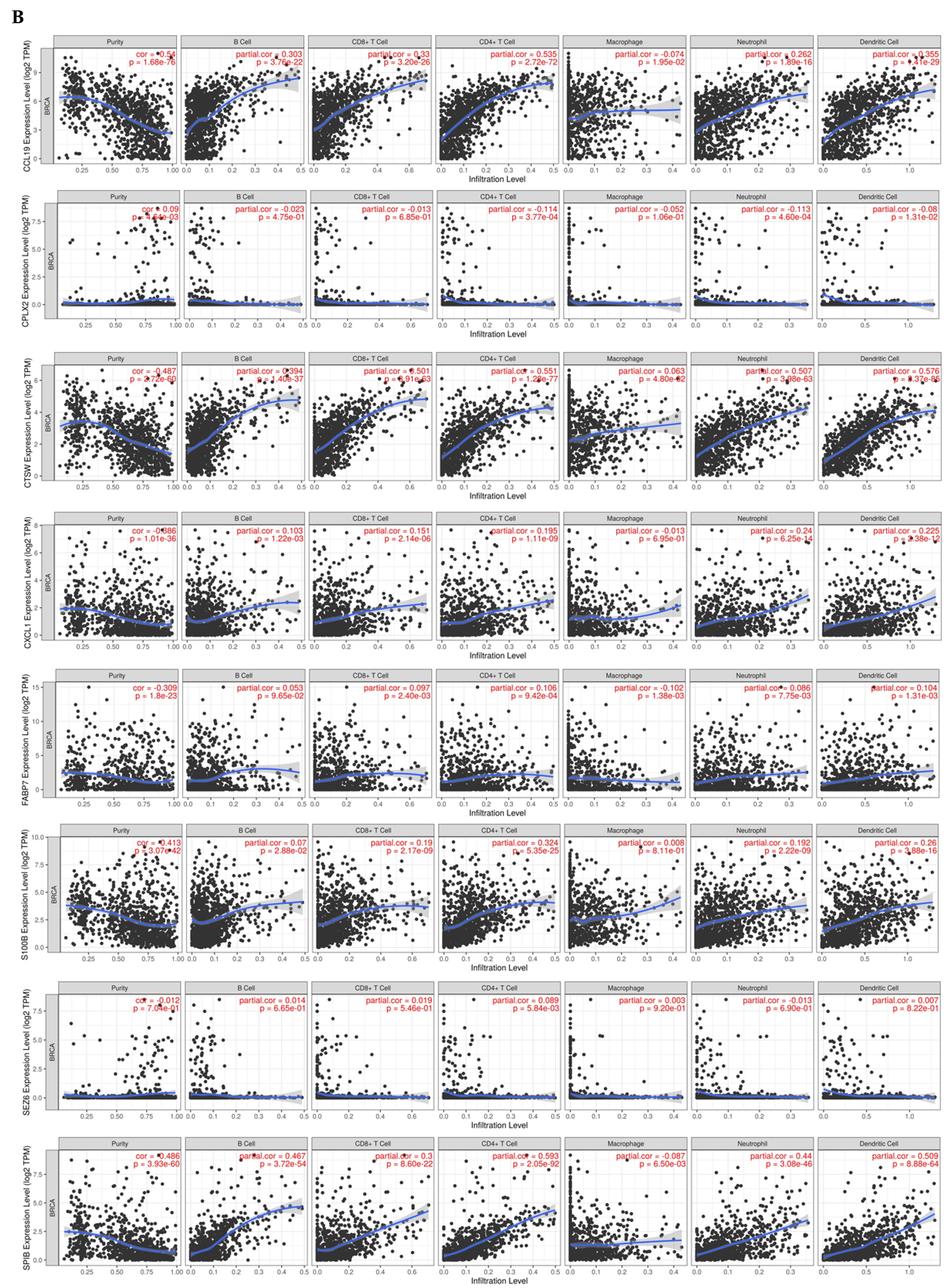

Supplement: Supplementary file 2 — Additional file 2: Figure S2. The relationship between key genes in the risk model and immune microenvironment. A Different types of SCNA of the key genes contributed to various enrichment in immune cells. B Correlation analyses between the expression level of the key genes and the immune cells. C Lollipop plots showing the correlation between immune cells and the expression of the key genes. D The expression of the key genes between normal and tumor tissue in various cancers. E The expression of the key genes between normal and tumor tissue in breast cancer in TCGA cohort. F The expression of the key genes between normal and tumor tissue in breast cancer in GEO cohort. G The protein expression of the key genes between normal and tumor tissue in breast cancer in HPA database. H The waterfall of the mutation landscape of the risk genes. I The summary plot showing the types of the gene mutation. [file 40001_2022_924_MOESM2_ESM.zip › Supplementary Figure 2B.jpg]

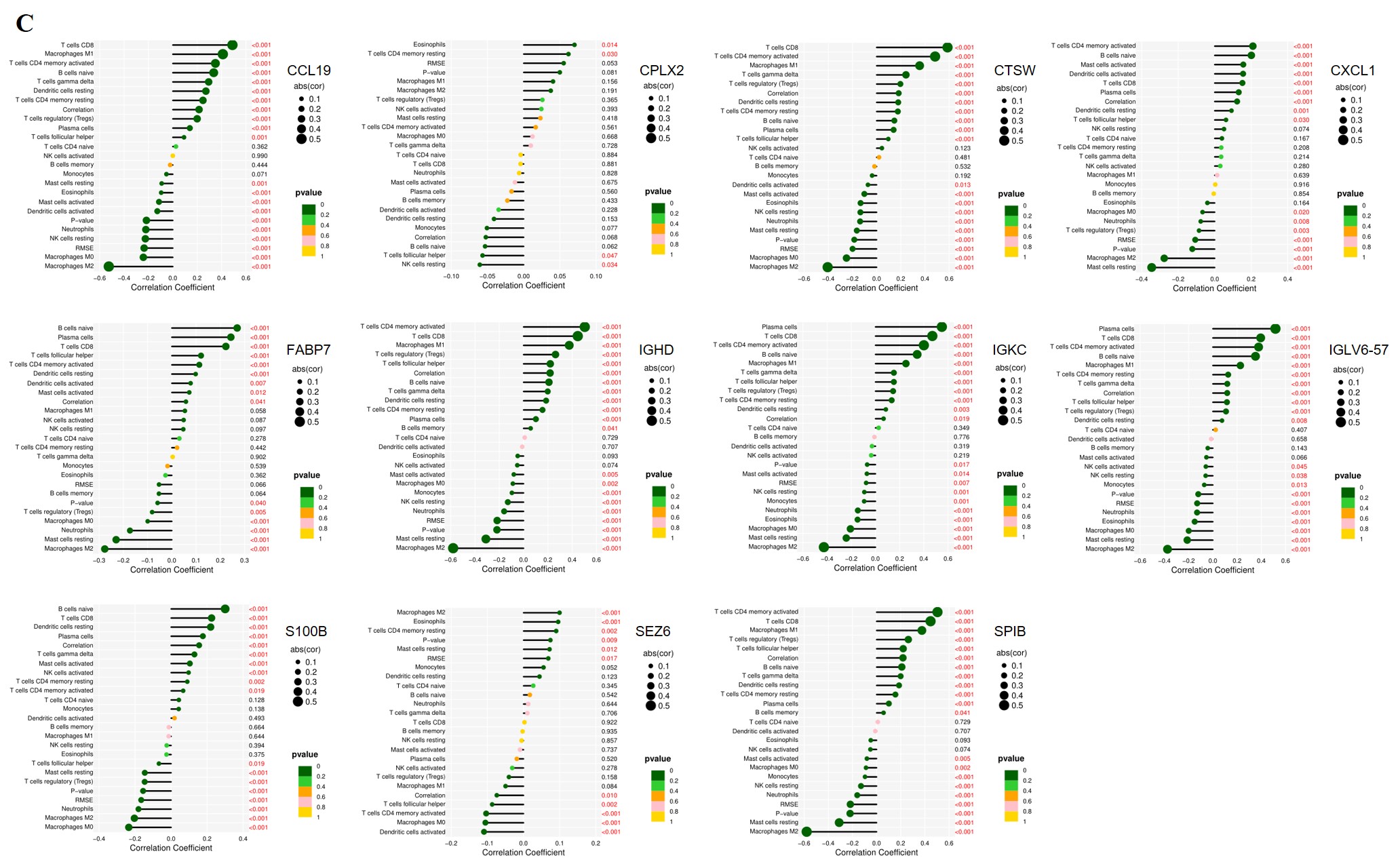

Supplement: Supplementary file 2 — Additional file 2: Figure S2. The relationship between key genes in the risk model and immune microenvironment. A Different types of SCNA of the key genes contributed to various enrichment in immune cells. B Correlation analyses between the expression level of the key genes and the immune cells. C Lollipop plots showing the correlation between immune cells and the expression of the key genes. D The expression of the key genes between normal and tumor tissue in various cancers. E The expression of the key genes between normal and tumor tissue in breast cancer in TCGA cohort. F The expression of the key genes between normal and tumor tissue in breast cancer in GEO cohort. G The protein expression of the key genes between normal and tumor tissue in breast cancer in HPA database. H The waterfall of the mutation landscape of the risk genes. I The summary plot showing the types of the gene mutation. [file 40001_2022_924_MOESM2_ESM.zip › Supplementary Figure 2C.jpg]

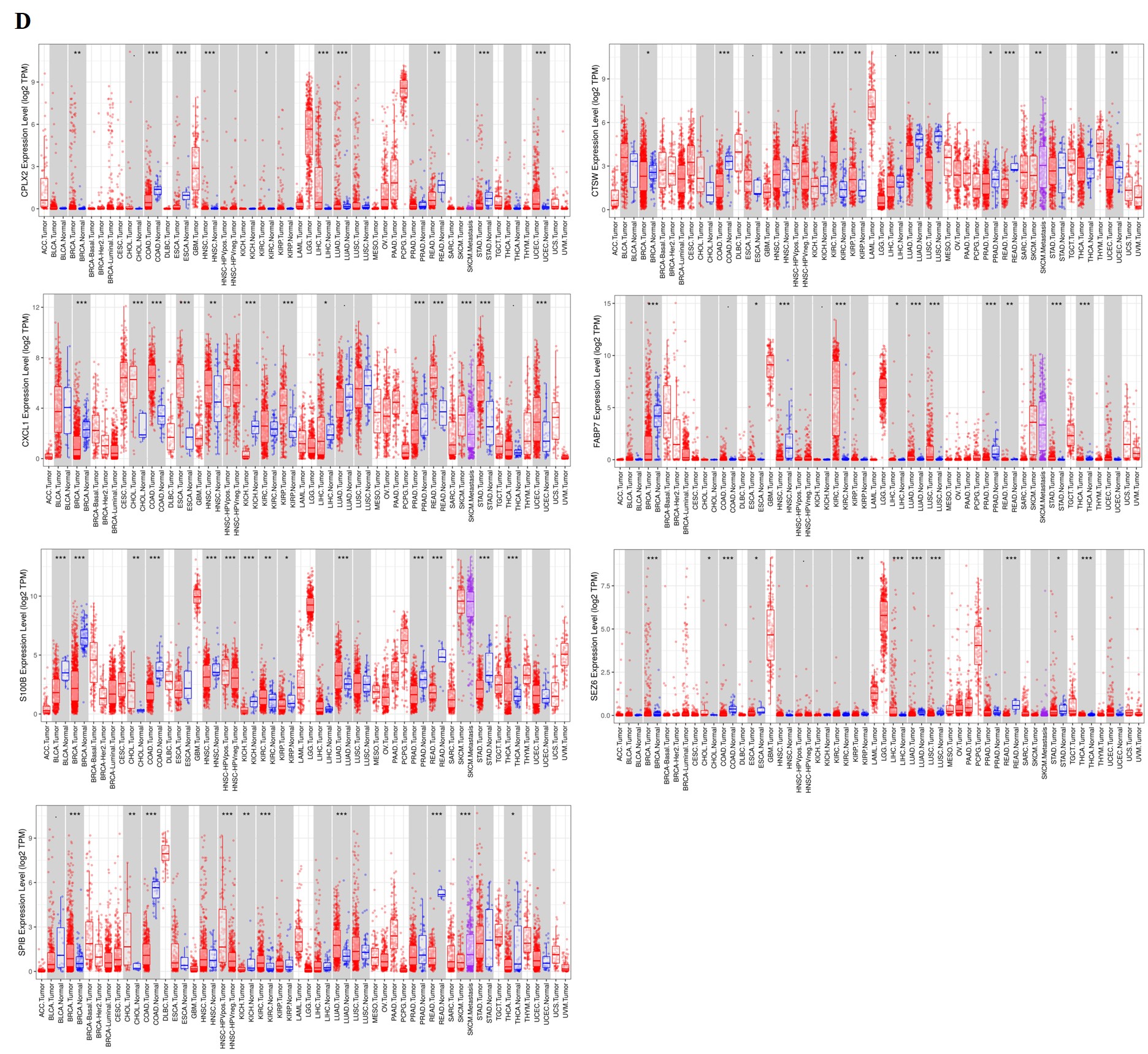

Supplement: Supplementary file 2 — Additional file 2: Figure S2. The relationship between key genes in the risk model and immune microenvironment. A Different types of SCNA of the key genes contributed to various enrichment in immune cells. B Correlation analyses between the expression level of the key genes and the immune cells. C Lollipop plots showing the correlation between immune cells and the expression of the key genes. D The expression of the key genes between normal and tumor tissue in various cancers. E The expression of the key genes between normal and tumor tissue in breast cancer in TCGA cohort. F The expression of the key genes between normal and tumor tissue in breast cancer in GEO cohort. G The protein expression of the key genes between normal and tumor tissue in breast cancer in HPA database. H The waterfall of the mutation landscape of the risk genes. I The summary plot showing the types of the gene mutation. [file 40001_2022_924_MOESM2_ESM.zip › Supplementary Figure 2D.jpg]

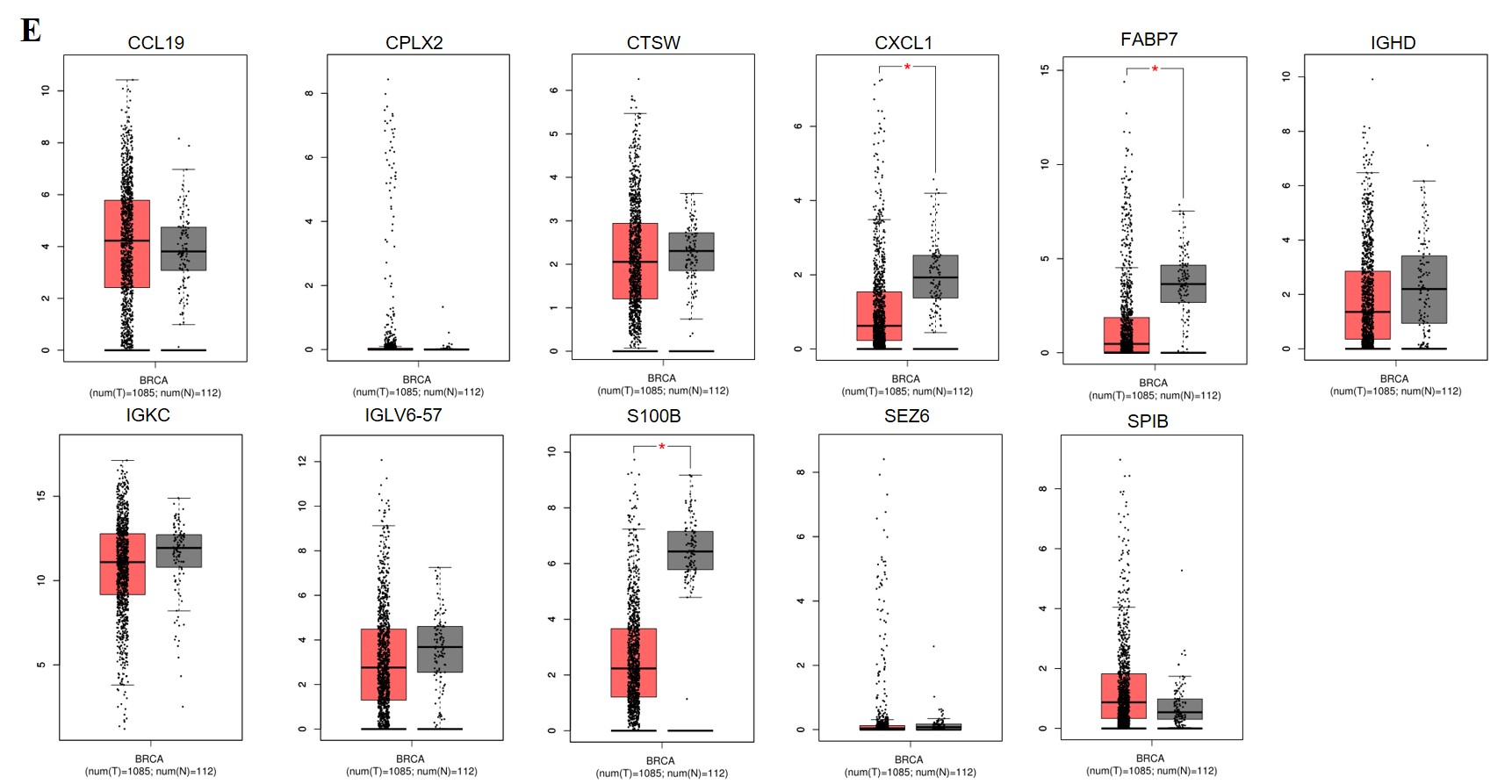

Supplement: Supplementary file 2 — Additional file 2: Figure S2. The relationship between key genes in the risk model and immune microenvironment. A Different types of SCNA of the key genes contributed to various enrichment in immune cells. B Correlation analyses between the expression level of the key genes and the immune cells. C Lollipop plots showing the correlation between immune cells and the expression of the key genes. D The expression of the key genes between normal and tumor tissue in various cancers. E The expression of the key genes between normal and tumor tissue in breast cancer in TCGA cohort. F The expression of the key genes between normal and tumor tissue in breast cancer in GEO cohort. G The protein expression of the key genes between normal and tumor tissue in breast cancer in HPA database. H The waterfall of the mutation landscape of the risk genes. I The summary plot showing the types of the gene mutation. [file 40001_2022_924_MOESM2_ESM.zip › Supplementary Figure 2E.jpg]

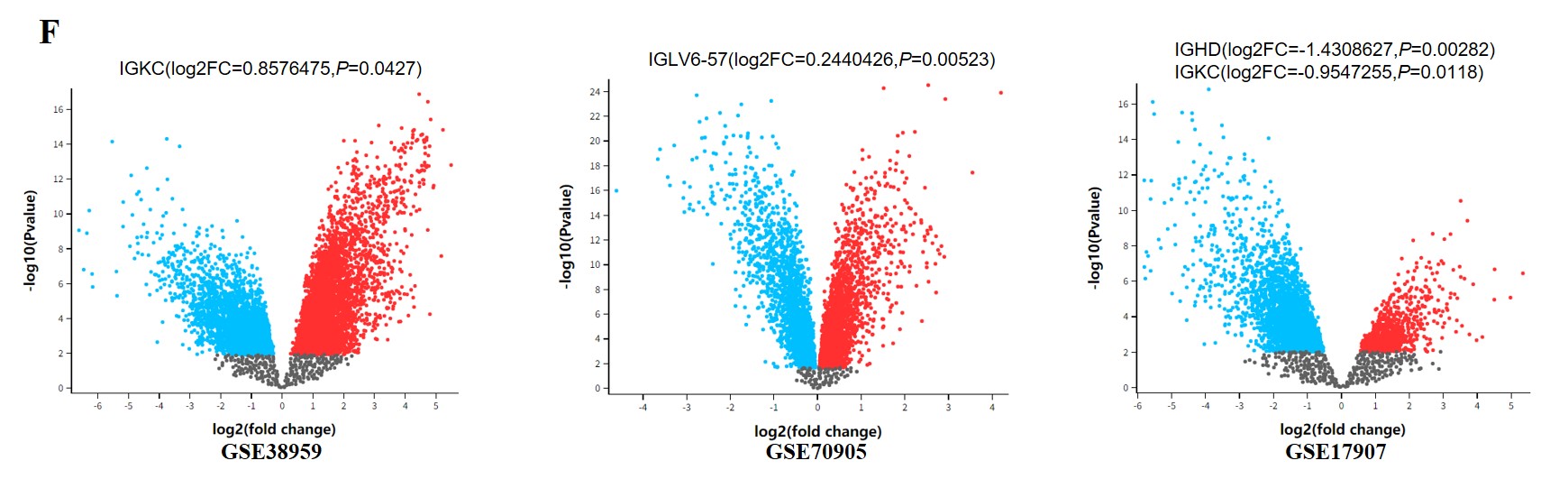

Supplement: Supplementary file 2 — Additional file 2: Figure S2. The relationship between key genes in the risk model and immune microenvironment. A Different types of SCNA of the key genes contributed to various enrichment in immune cells. B Correlation analyses between the expression level of the key genes and the immune cells. C Lollipop plots showing the correlation between immune cells and the expression of the key genes. D The expression of the key genes between normal and tumor tissue in various cancers. E The expression of the key genes between normal and tumor tissue in breast cancer in TCGA cohort. F The expression of the key genes between normal and tumor tissue in breast cancer in GEO cohort. G The protein expression of the key genes between normal and tumor tissue in breast cancer in HPA database. H The waterfall of the mutation landscape of the risk genes. I The summary plot showing the types of the gene mutation. [file 40001_2022_924_MOESM2_ESM.zip › Supplementary Figure 2F.jpg]

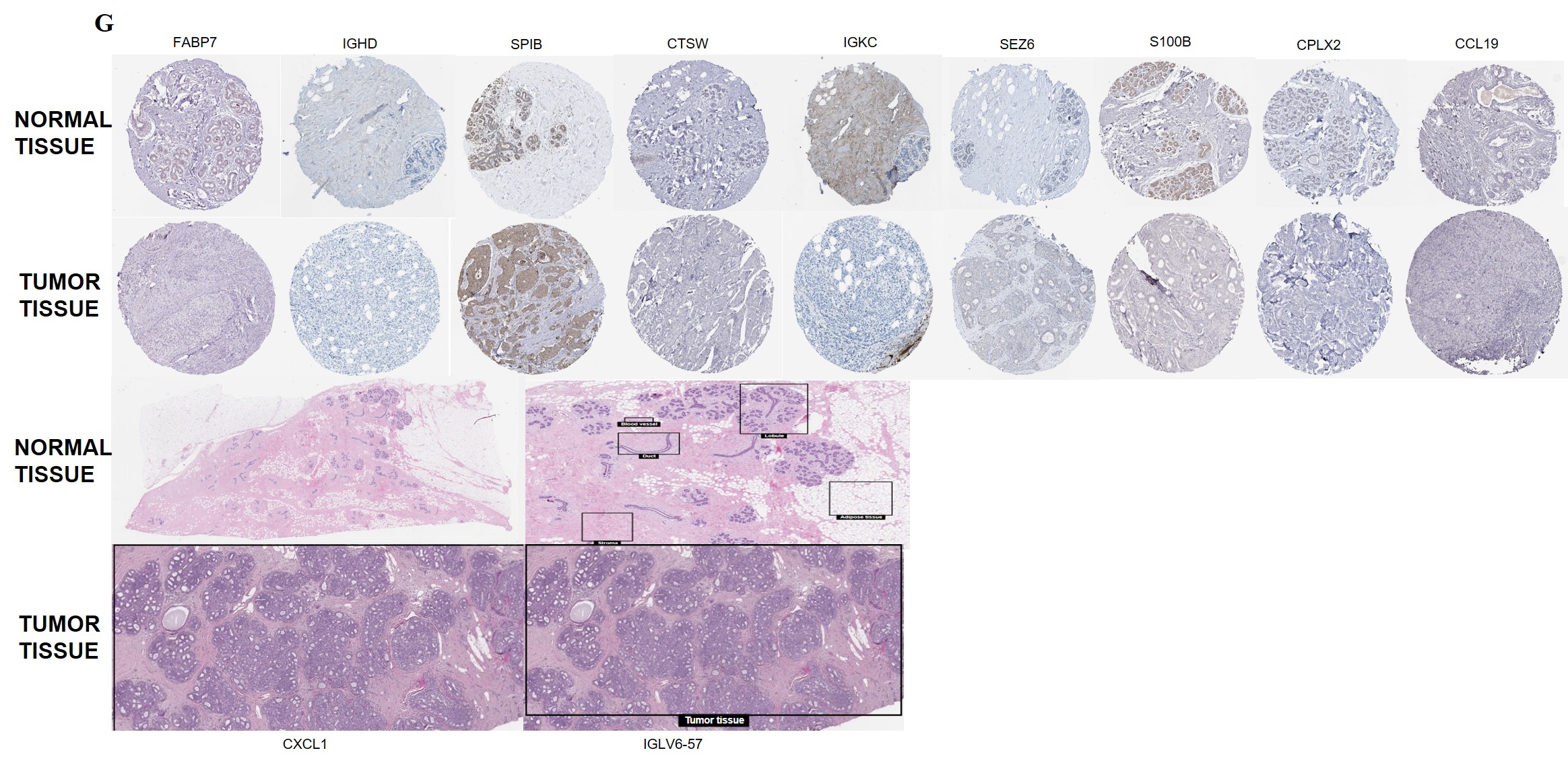

Supplement: Supplementary file 2 — Additional file 2: Figure S2. The relationship between key genes in the risk model and immune microenvironment. A Different types of SCNA of the key genes contributed to various enrichment in immune cells. B Correlation analyses between the expression level of the key genes and the immune cells. C Lollipop plots showing the correlation between immune cells and the expression of the key genes. D The expression of the key genes between normal and tumor tissue in various cancers. E The expression of the key genes between normal and tumor tissue in breast cancer in TCGA cohort. F The expression of the key genes between normal and tumor tissue in breast cancer in GEO cohort. G The protein expression of the key genes between normal and tumor tissue in breast cancer in HPA database. H The waterfall of the mutation landscape of the risk genes. I The summary plot showing the types of the gene mutation. [file 40001_2022_924_MOESM2_ESM.zip › Supplementary Figure 2G.jpg]

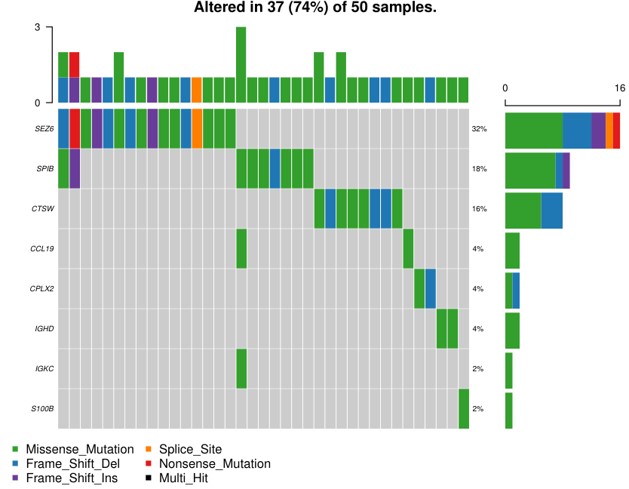

Supplement: Supplementary file 2 — Additional file 2: Figure S2. The relationship between key genes in the risk model and immune microenvironment. A Different types of SCNA of the key genes contributed to various enrichment in immune cells. B Correlation analyses between the expression level of the key genes and the immune cells. C Lollipop plots showing the correlation between immune cells and the expression of the key genes. D The expression of the key genes between normal and tumor tissue in various cancers. E The expression of the key genes between normal and tumor tissue in breast cancer in TCGA cohort. F The expression of the key genes between normal and tumor tissue in breast cancer in GEO cohort. G The protein expression of the key genes between normal and tumor tissue in breast cancer in HPA database. H The waterfall of the mutation landscape of the risk genes. I The summary plot showing the types of the gene mutation. [file 40001_2022_924_MOESM2_ESM.zip › Supplementary Figure 2H.jpg]

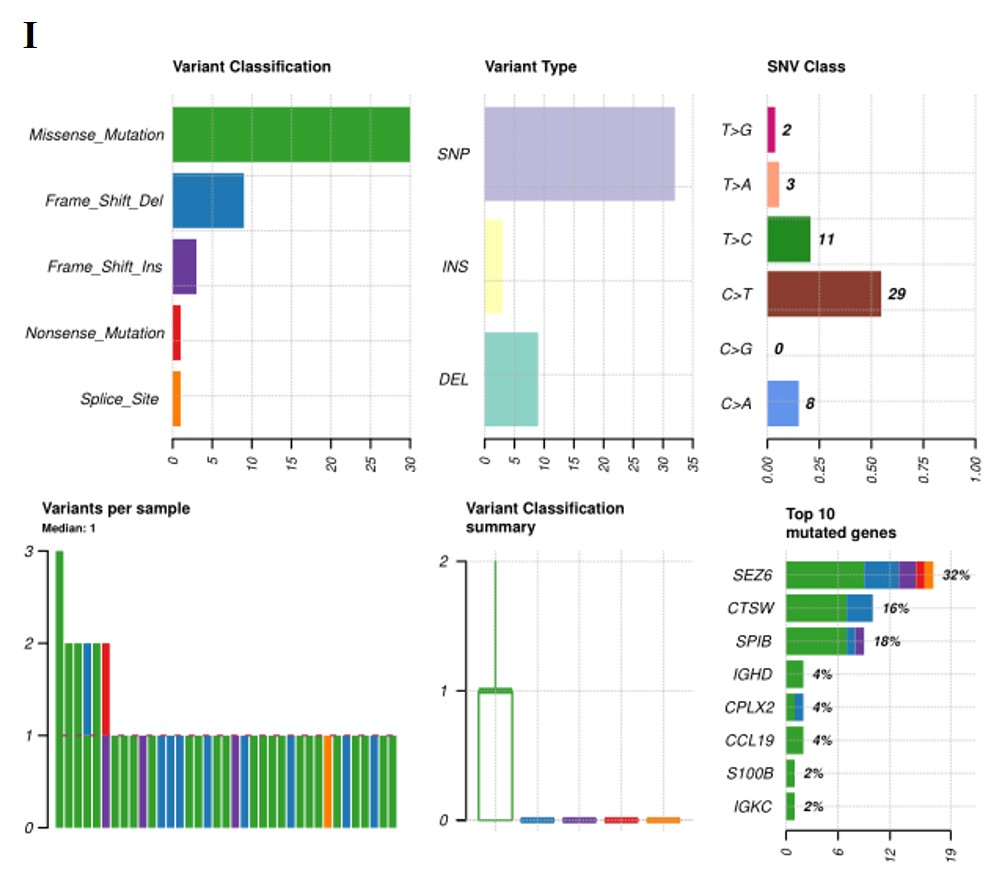

Supplement: Supplementary file 2 — Additional file 2: Figure S2. The relationship between key genes in the risk model and immune microenvironment. A Different types of SCNA of the key genes contributed to various enrichment in immune cells. B Correlation analyses between the expression level of the key genes and the immune cells. C Lollipop plots showing the correlation between immune cells and the expression of the key genes. D The expression of the key genes between normal and tumor tissue in various cancers. E The expression of the key genes between normal and tumor tissue in breast cancer in TCGA cohort. F The expression of the key genes between normal and tumor tissue in breast cancer in GEO cohort. G The protein expression of the key genes between normal and tumor tissue in breast cancer in HPA database. H The waterfall of the mutation landscape of the risk genes. I The summary plot showing the types of the gene mutation. [file 40001_2022_924_MOESM2_ESM.zip › Supplementary Figure 2I.jpg]

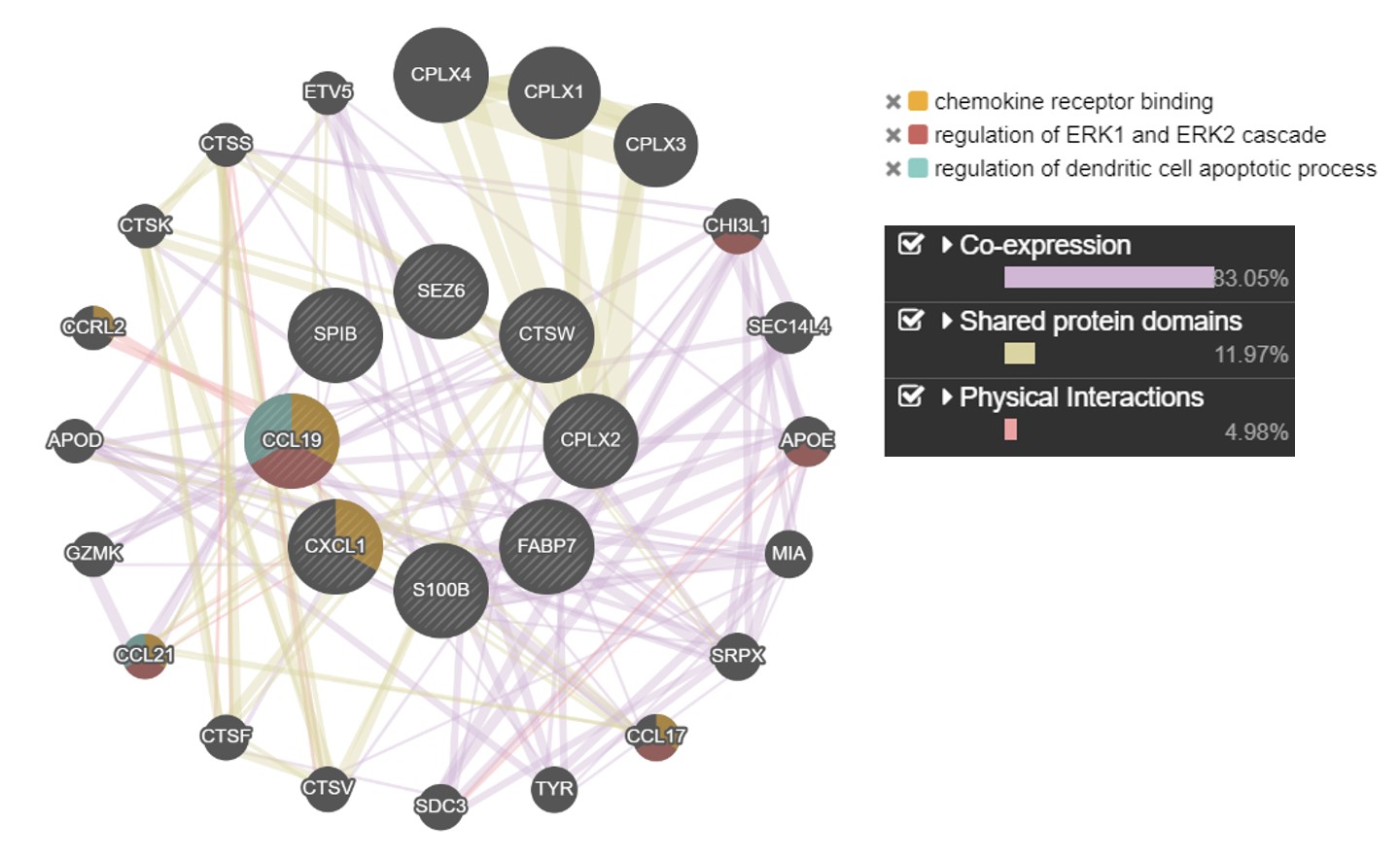

Supplement: Supplementary file 3 — Additional file 3: Figure S3. PPI network to demonstrate the interactions of the risk genes at the GeneMANIA. [file 40001_2022_924_MOESM3_ESM.jpg]
